# Desensitizing Mitochondrial Permeability Transition by ERK-Cyclophilin D Axis Contributes to the Neuroprotective Effect of Gallic Acid against Cerebral Ischemia/Reperfusion Injury

**DOI:** 10.3389/fphar.2017.00184

**Published:** 2017-04-06

**Authors:** Jing Sun, Da-Dui Ren, Jin-Yi Wan, Chen Chen, Dong Chen, Huan Yang, Chun-Lai Feng, Jing Gao

**Affiliations:** ^1^Neurobiology and Mitochondrial Key Laboratory, School of Pharmacy, Jiangsu UniversityZhenjiang, China; ^2^Department of Traditional Chinese Medicine, School of Pharmacy, Jiangsu UniversityZhenjiang, China; ^3^Department of Pharmaceutics, School of Pharmacy, Jiangsu UniversityZhenjiang, China

**Keywords:** gallic acid (GA), mitochondrial permeability transition pore (MPTP), cyclophilin D (CypD), extracellular signal-regulated kinases (ERK), cerebral ischemia/reperfusion

## Abstract

Ischemic stroke is a devastating disease with complex pathophysiology. Much evidence confirms that opening of the mitochondrial permeability transition pore (MPTP) is related with mitochondrial dysfunction to apoptosis in ischemic stroke, thus elucidating its signaling mechanism and screening novel MPTP inhibitor is therefore of paramount importance. Our earlier studies identified that gallic acid (GA), a naturally occurring plant phenol, endows with effect on inhibition of mitochondrial dysfunction, which has significant neuroprotective effect in cerebral ischemia/reperfusion injury. However, its molecular mechanisms regulating mitochondrial dysfunction remain elusive. Here, we uncover a role of GA in protecting mitochondria via MPTP inhibition. In addition to inhibit CypD binding to adenine nucleotide translocator, GA potentiates extracellular signal-regulated kinases (ERK) phosphorylation, leading to a decrease in cyclophilin D (CypD) expression, resulting in a desensitization to induction of MPTP, thus inhibiting caspase activation and ultimately giving rise to cellular survival. Our study firstly identifies ERK-CypD axis is one of the cornerstones of the cell death pathways following ischemic stroke, and confirms GA is a novel inhibitor of MPTP, which inhibits apoptosis depending on regulating the ERK-CypD axis.

## Introduction

Ischemic stroke is a major cause of adult disability and ranks only behind cancer and cardiac disease as cause of death (Flynn et al., 2008). As our current understanding of cerebral ischemia injury is greatly contributed by the notion that mitochondria are both a pivotal site for cell death through mitochondrial apoptotic pathways (Andreux et al., 2013) and a central target of processes triggered by cerebral ischemia/reperfusion, such as elevation in intracellular Ca^2+^ (Sims and Anderson, 2002) and reactive oxygen species (ROS) (Sanderson et al., 2013), it is not surprising that strategies aimed at protecting against cerebral ischemia/reperfusion damage have focused on mitochondria.

Mitochondria have a central role in cell death including apoptosis, necrosis, and necroptosis via mitochondrial membrane permeabilization (Kroemer et al., 2007; Zhang et al., 2016). In response to pro-apoptotic stimuli, including ROS and Ca^2+^ overload, the inner mitochondrial membrane abrupt loss of permeability to small solutes (Criddle et al., 2006; Baumgartner et al., 2009). This phenomenon, which is known as mitochondrial permeability transition (MPT), results in dissipation of the MMP, release of apoptotic factors, osmotic swelling of the mitochondrial matrix, and eventually cell death (Fulda et al., 2010; Vanden Berghe et al., 2014).

The MPT is caused by the opening of a non-selective pore assembled at the junctions between the inner and outer mitochondrial membrane, the so-called ‘MPT pore’ (MPTP) (Fulda et al., 2010). While a large number of studies pointed to various proteins such as VDAC, ANT, and F_1_F_O_ ATP-synthase complexes as to structural or regulatory MPTP components, however, the precise molecular mechanisms of MPTP remained a matter of debate until now (Kokoszka et al., 2004; Baines et al., 2007; Bonora et al., 2015). As a standalone exception, CypD has been ascribed an essential regulatory role in MPTP via robust genetic studies as early as in 2005 (Baines et al., 2005; Schinzel et al., 2005; Vaseva et al., 2012).

Cyclophilin D is a *ppif* gene product, a member of the immunophilin family of peptidylprolyl *cis-trans* isomerases (Galat and Metcalfe, 1995), which is well known bind to mitochondrial ANT, thus promoting MPTP opening (Eliseev et al., 2009; Fulda et al., 2010). Indeed, as a chaperone protein, CypD promotes the rearrangement of MPTP subunits to allow for channel formation (Galat and Metcalfe, 1995; Eliseev et al., 2009; Bonora and Pinton, 2014). Recently study characterizes CypD-F_1_F_O_ ATP-synthase interaction from the point of view of MPTP formation, providing evidence that CypD targets both energy production and programmed cell death (Elrod and Molkentin, 2013).

In line with this notion, the *Ppif^-/-^* genotype as well as the systemic administration of chemical CypD inhibitors such as CsA confer remarkable protection in animal models of ischemia/reperfusion injury *in vivo*, and resistant to H_2_O_2_-induced cell death *in vitro* (Schinzel et al., 2005; Hu et al., 2010; Vaseva et al., 2012; Cho et al., 2013). In addition to CsA and its derivatives, barely drugs have been validated as compounds specifically targeting CypD or functions of MPTP (Muramatsu et al., 2007; Martin et al., 2014). What is more, the serious adverse reactions of these compounds limit the clinical application on cardiovascular diseases due to their immunosuppressive effects (Rezzani, 2004).

**FIGURE 1:**
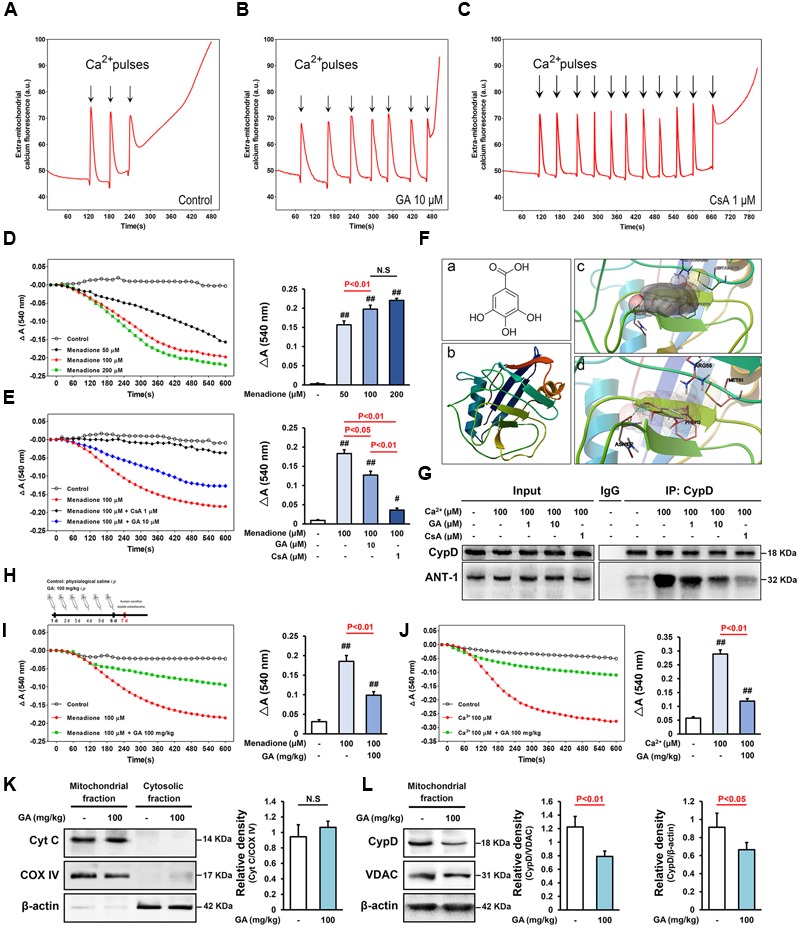
**Gallic acid (GA) desensitizes MPTP via a signaling axis that involves CypD in liver mitochondria.** CRC was determined by the concentration of calcium required to trigger MPTP opening.**(A,B)** GA has a higher Ca^2+^ threshold than control mitochondria. **(C)** CsA (1 μM) robustly increases the number of spikes, consistent with the importance of MPTP in this process. **(D)** Menadione caused marked mitochondria swelling in a concentration-dependent manner, **(E)** whereas GA or CsA pre-incubated mitochondria were significantly less sensitive. Histogram comparing Δ*A*_540_ values (*A*_540__max_–*A*_540__min_) among groups (*n* = 10). **(F-a)** Chemical structure of GA. **(F-b)** The 3D structure of CypD obtained from Protein Data Bank (PDB ID: 2BIT). **(F-c)** Salt bridges to ASN 102, PHE 113, MET 61, ARG 55, and **(F-d)** an H-bond to ASN 102 make major contributions to the binding affinity for GA (Distance: 2.039, Estimated free energy of binding: –0.568). **(G)** Representative Immunoprecipitation analysis showed that Ca^2+^-induced increase of CypD binding to ANT-1 was blocked by CsA or GA (*n* = 6). GA desensitized liver mitochondria to the permeability transition via suppressing CypD expression. **(H)** Mitochondria isolated from mouse liver which pre-treatment with GA (100 mg/kg) once a day for 6 days. The level of mitochondrial swelling triggered by menadione **(I)** or Ca^2+^ **(J)** was significantly decreased following GA pre-treatment (*n* = 10). The release of Cyto C **(K)**, and the expression of CypD **(L)** were tested via Western Blotting (*n* = 6). COX IV, VDAC, and β-actin were used as loading controls. Data reported as the means ± SD. *P* values were obtained using two-way analysis of variance (ANOVA) test. N.S. indicates a *P* value > 0.05. ^##^*P* < 0.01, ^#^*P* < 0.05 versus control group.

GA (3, 4, 5-trihydroxybenzoic acid, **Figure 1F-a**), an important plant-sourced polyphenolic compound (Shen et al., 2013), reduced non-steroidal anti-inflammatory drugs (NSAID)-induced gastropathy, and radiation cytotoxicity by blocking oxidative stress-mediated apoptosis (Pal et al., 2010; Timiri Shanmugam et al., 2016). Our earlier studies identified that GA is a potential mitochondrial protective agent, which has significant neuroprotective effect in cerebral ischemia/reperfusion injury (Sun et al., 2014). Nevertheless, its mitochondrial protective mechanism remains unclear.

Here, we reveal a role of GA in protecting mitochondria via MPTP inhibition. To our surprise, we found that GA not only prevented MPTP opening by specifically interacted with CypD, but also played a role in regulating CypD expression through activating extracellular regulated protein kinases (ERK)-dependent phosphorylation, which renders nerve cells more refractory to MPTP opening following H_2_O_2_ (a generator of oxidative stress) *in vitro* or MCAO injury *in vivo*. These results confirm GA is a novel inhibitor of MPTP, which inhibits apoptosis depending on regulating the ERK-CypD axis. To the best of our knowledge this is the first study describing a novel MPTP inhibitor with pharmacological effect on CypD expression.

## Materials and Methods

### Chemicals, Preparation of Mitochondria, Docking, and Experimental Design

Please see Supplementary Material.

### Animals and Ethics Statement

Adult male C57BL/6 mice (18–22 g) and Sprague-Dawley rats (250–300 g) were purchased from Jiangsu University Laboratory Animals Center, Zhenjiang, China. All animal procedures used in this study were approved by the Ethics Committee for Animal Experimentation of Jiangsu University (UJS-20160012, in Supplementary Material). The drug treatment protocols of GA, CsA, and U0126 in this experiment were based on previous literature (Maddahi and Edvinsson, 2008; Cho et al., 2013; Sun et al., 2014).

### Mitochondrial Calcium Retention Capacity (CRC)

Mitochondrial CRC was assessed fluorimetrically in the presence of the fluorescent Ca^2+^ indicator Calcium Green 5N by the QuantaMaster & TimeMaster Spectrofluorometer (Photon Technology International, QuantaMaster^TM^40, USA) at excitation/emission maxima of 505/535 nm. Mitochondria (0.5 mg) were diluted in CRC buffer (120 mM KCl, 10 mM NaCl, 1 mM KH_2_PO_4_, 10 mM MOPS, 20 mM HEPES-Tris, pH 7.2) containing 150 nM Calcium Green 5N. Mitochondria were pulse-loaded with CaCl_2_ (10 μM each). Compounds were added to the mitochondrial suspension 5 min before CaCl_2_ exposure. The ratio between the amounts of calcium required to trigger MPTP in the presence of the compound (CRC_i_) with respect to that required to induce MPTP in the absence of the compound (CRC_o_) was used for statistical analysis.

### Mitochondrial Swelling

Mitochondrial swelling assay buffer (120 mmol/L KCl, 10 mmol/L Tris-HCl, 20 mmol/L MOPS, 5 mmol/L KH_2_PO_4_, pH 7.4) containing mitochondrial solution at 1 mg/ml was placed in each well of a 96-well plate at 25°C. The swelling was measured with a spectrophotometer (Molecular Device, Spectra Max 190, USA) as a decrease in light scattering at 540 nm for 10 min. Compounds were added to the mitochondrial suspension 5 min before CaCl_2_ or menadione exposure. The difference (ΔA_540_) between the maximum A_540_ value (immediately after CaCl_2_ or menadione addition) and the minimum A_540_ value (10 min after CaCl_2_ or menadione addition) were used for statistical analysis.

### Immunoprecipitation

After pre-treatment with compounds for 5 min and 100 μM CaCl_2_ exposured to 10 min, the isolated liver mitochondria were lysed in PBS containing 1% TritonX-100 and protease inhibitors cocktail. The supernatant was precleared with Protein A/G beads for 2 h at 4°C, and 500 mg of precleared protein extracts were incubated with 1 μg of anti-CypD antibody or normal rabbit IgG overnight at 4°C. The precipitated complexes were washed in lysis buffer and bound proteins analyzed by immunoblots.

### Western Blotting

Samples were prepared to isolation of mitochondria and cytosolic protein by a mitochondria isolation kit according to the manufacturer’s guidelines. Equal amounts of proteins were resolved by SDS/polyacrylamide gel electrophoresis (PAGE) using 10–15% acrylamide, transferred onto polyvinylidene difluoride (PVDF) membranes, and blotted using the following commercially available antibodies: anti-CypD (1:500), anti-ANT (1:300), anti-VDAC (1:400), anti-Cyto C (1:300), anti-Caspase-3 (1:500), anti-Caspase-8 (1:300), anti-Cleaved-Caspase-9 (1:400), anti-ERK1/2 (1:500), and anti-p-ERK1/2 (1:500), anti-β-actin (1:4000), anti-COX IV (1:500) followed by incubation with the corresponding horseradish peroxidase-labeled secondary antibody (1:2000). Protein bands were visualized and analyzed using the Chemifluorescence Imaging Systems (GE, USA).

### Induction of Transient Cerebral Ischemia

Transient cerebral ischemia was induced by MCAO according to the method of Longa et al. (1989). The rats were anesthetized with chloral hydrate (350 mg/kg intraperitoneal injection). To alleviate pain, animals received 0.05 mg/kg subcutaneous buprenorphine immediately after the reperfusion anesthesia. The level of rCBF was achieved using a laser-Doppler flowmeter (Moor Instruments, UK). Rats had a rCBF in 20% of the pre-ischemic baseline value 10 min after ischemia and to the 80% of baseline at 30 min after reperfusion were used in this study (Mao et al., 2015). Physiological parameters such as the mean arterial blood pressure and heart rate were all monitored by Power Lab System (AD Instruments, Australia). The rats in the sham operation group underwent vessel exposure without MCAO. Body temperature was regulated at 37.0 ± 0.5°C with a heating pad and lamp when necessary. The details of the exclusion criteria, the level of rCBF, and physiological parameters were shown in the Supplementary Material.

### Assessment of Cerebral Infarct Volume

2,3,5-Triphenyl-tetrazolium chloride staining was carried out using methods previously published by Leker et al. (2003). Brain Infarct volumes were calculated according to the following formula: (contralateral volume-undamaged ipsilateral)/contralateral volume (%).

### Histopathological Analysis

Rat brains were removed, fixed in 4% paraformaldehyde in phosphate-buffered saline for 72 h at room temperature, dehydrated through a graded ethanol series, and embedded in paraffin. Sections of 4–5 μm thick were cut in the coronal plane and stained with H & E staining, which was performed as previously described (Monaghan et al., 2016).

### Immunostaining

Immunostaining methods were used as described previously (Fernandez-Godino et al., 2016). The following primary antibodies were used: anti-Cyto C (1:150), anti-Cleaved-Caspase-9 (1:100), and anti-NeuN (1:200). Following incubation with the primary antibody at 4°C overnight, sections were incubated for 90 min with Alexa Fluor 594-and Alexa Fluor 488-conjugated secondary antibodies (1:200) in blocking solution at room temperature. Finally, sections were incubated with DAPI for 10 min to detect nuclei. All the slices were visualized by fluorescent inverted microscope (Nikon, Ti-E Live Cell Imaging System, Japan).

### Electron Microscopy

Rats brains were removed, dissected, and the ipsilateral penumbra areas post-fixed in the same fixative for 2 h before transferring to 0.12 M PBS. Specimens were immersed in 1% OsO_4_ in cacodylate buffer, dehydrated in ethanol and embedded in epoxy resin. Ultrathin sections (60 nm) were obtained with an ultra microtome (Leica EM UC7) and then were stained with uranyl acetate and lead citrate for examination with a transmission electron microscope (TEM) at 80 kV (HITACHI, H-7650, Japan).

### Cell Cultures and Treatments

Human neuroblastoma SH-SY5Y cells were cultured in 90% modified Eagle’s medium-F12 medium and supplemented with 10% fetal bovine serum. All cultures were maintained in 10 cm tissue culture dishes to approximately 90% confluence in a humidified atmosphere of 5% CO_2_-95% air at 37°C. The SH-SY5Y cells were treated with H_2_O_2_ at concentration of 500 μM for 4 h to establish the MPTP opening. Various concentrations of GA (0.1, 1, 10 μM), EGF (10, 100 ng), and CsA (1 μM) were added into the cultures 24 h before the onset of H_2_O_2_-inducing MPTP opening. Cells were incubated with U0126 (1, 10 μM) in culture medium for 2 h before GA or EGF treatment, which was used to inhibit ERK phosphorylation as recommended by the manufacturer.

### Plasmid Transfection

Clustered regularly interspaced short palindromic repeats-CypD plasmid synthesized by Santa Cruz Biotechnology was transfected into SH-SY5Y cells using the UltraCruz Transfection Reagent. Samples were collected at 24 and 48 h, and the transfection efficiency was examined by Western bolt. According to the manufacturer’s instructions and our experiment data, the cells were used following 48 h transfection. Control cells were treated control CRISPR activation plasmid in an identical way.

### Cell Viability and Apoptosis

Cell viability was measured by MTT assay (Ganot et al., 2013). The optical density was spectrophotometrically measured at 570 nm using a microplate reader (Molecular Device, Spectra Max 190, USA) with DMSO as the blank. Cell apoptosis was determined by the Annexin V-FITC/PI apoptosis detection kit. A total of 20,000 cells of each sample were analyzed by bivariate flow cytometry system (Becton-Dickinson, FACSCanto II, USA).

### Assaying MPTP Opening in Live Cells

To obtain direct evidence of GA effect on MPTP, the Calcein AM-CoCl_2_ assay was performed as previously described (Bonora et al., 2016). The results were visualized using fluorescent inverted microscope (Nikon, Ti-E Live Cell Imaging System, Japan). Alternatively, fluorescence intensity was monitored using flow cytometry (Becton-Dickinson, FACSCanto II, USA) at excitation/emission maxima of 490/515 nm.

### Assessment of Mitochondrial Membrane Potential (MMP)

A cationic dye, JC-1, was used to monitor the MMP. Briefly, cells were treated JC-1 10 μM for 30 min incubation at 37°C. The results were visualized using fluorescent inverted microscope (Nikon, Ti-E Live Cell Imaging System, Japan). Alternatively, fluorescence intensity was monitored using flow cytometry (Becton-Dickinson, FACSCanto II, USA) at excitation/emission maxima of 585/590 nm (J-aggregates) and 514/529 nm (monomer).

### Statistical Analyses

Except neurological deficit scores, all values are expressed as the mean ± SD, and the statistical significance of difference between groups was determined by a two-way analysis of variance (ANOVA) test. Neurological deficit scores were expressed as the median, and the statistical significance of the difference between groups was determined by a non-parametric Mann Whitney test. A *P*-value < 0.05 was considered to indicate statistical significance.

## Results

### GA Inhibits MPTP in Liver Mitochondria in both Ca^2+^ and ROS Stimuli

Matrix Ca^2+^ is the single most important factor for inducing MPTP (Baumgartner et al., 2009; Fulda et al., 2010). Here, mitochondrial CRC was assessed fluorimetrically in the presence of the fluorescent Ca^2+^ indicator Calcium Green 5N. As shown in **Figure 1**, control mitochondria buffered 30 μM CaCl_2_, and their buffering capacity was significantly increased after pre-incubation with 1 μM CsA, consistent with the importance of MPTP in this process (**Figures 1A,C** and Supplementary Table 1; *n* = 10, *P* < 0.01). As expected, mitochondria that pre-incubation with 10 μM GA buffered up to 60 μM CaCl_2_, strongly increased the level of CRC as observed in control mitochondria (**Figures 1A,B** and Supplementary Table 1; *n* = 10, *P* < 0.01).

We further assessed MPTP sensitivity to the oxidant menadione, which acts as a potent MPTP inducer through promoting oxidative stress (Criddle et al., 2006). The ΔA_540_ value of the mitochondria exposed to menadione at concentrations of 50–200 μM for 10 min increased to 0.1566 ± 0.01, 0.1975 ± 0.02, and 0.2203 ± 0.01 of the control value, respectively (**Figure 1D**; *n* = 10). Menadione caused marked mitochondria swelling, whereas CsA pre-incubated mitochondria were significantly less sensitive (**Figure 1E**; *n* = 10, *P* < 0.01). Importantly, GA prevent mitochondrial swelling induced by stimuli independent of calcium overload (**Figure 1E**; *n* = 10, *P* < 0.05), suggesting that it act as a genuine inhibitor of MPTP and not by affecting the calcium homeostasis.

### GA Protects Brain Mitochondria against Cerebral Ischemia Reperfusion Injury

**FIGURE 2:**
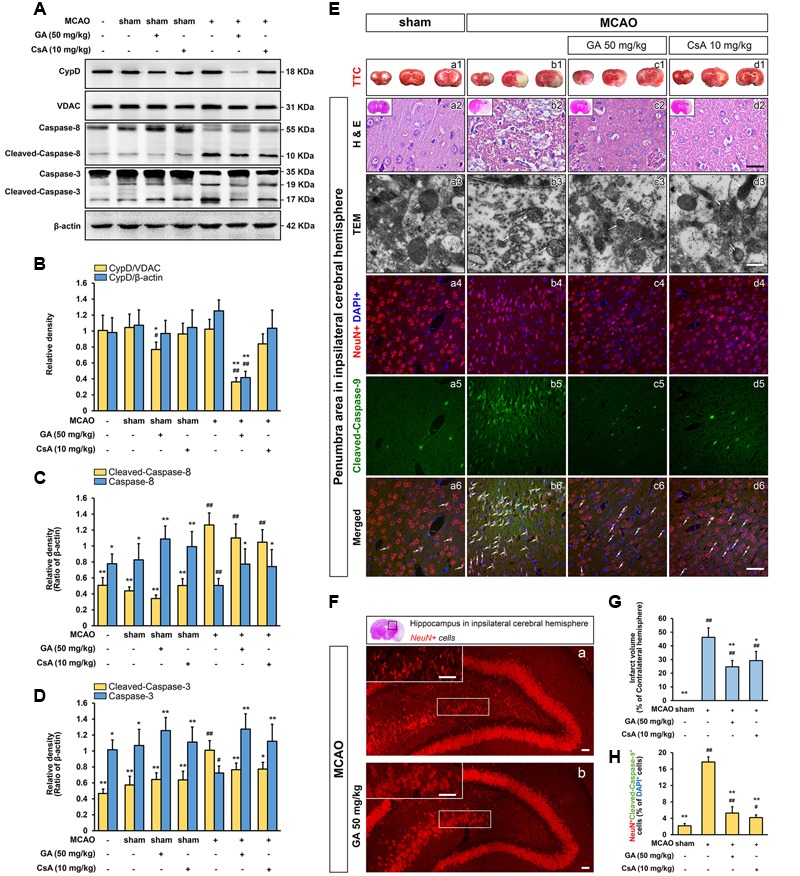
**Gallic acid protects neuron against MCAO insult by the mitochondrial-dependent pathway.** GA downregulated brain mitochondrial CypD, inhibited the expression of Cleaved-caspase-8, and Cleaved-caspase-3 protein levels in brain mitochondria following transient cerebral ischemia (*n* = 4; **A–D**). VDAC, and β-actin were used as loading controls. GA protected brain mitochondrial and confers neuron survival after transient cerebral ischemia. **(E-a1–d1)** Representative TTC-stained coronal brain sections; white indicates infarcted tissue. **(E-a2–d2)** Representative H & E stained coronal brain sections (*n* = 4). Scale bars, 10 μm. **(E-a3–d3)** The TEM results evidence that GA protected brain mitochondria against MCAO injury (*n* = 4). Scale bars, 1 μm. **(E-a4**–**6, b4**–**6, c4**–**6, d4**–**6)** Immunohistochemistry depicting colocalization of Cleaved-caspase-9 with NeuN following 48 h reperfusion. Scale bars, 10 μm. **(F)** The numbers of pyramidal neurons in the hippocampus region were also diminished by Immunohistochemistry. Scale bars, 10 μm. **(G)** Histogram showing the infarct volume (% of contralateral hemisphere) in TTC-stained brain sections (*n* = 8). **(H)** Histogram showing the percentage of NeuN and Cleaved-caspase-9-positive cells which described as the number of positive cells/total number of cells (*n* = 4). Data reported as the means ± SD. *P* values were obtained using two-way analysis of variance (ANOVA) test. ^##^*P* < 0.01, ^#^*P* < 0.05 versus sham group. ^∗∗^*P* < 0.01, ^∗^*P* < 0.05 versus MCAO group.

Next, we are interested in the MPTP activity of GA in cerebral ischemia insult, which is well known to favor activation of MPTP *in vivo* (Sun et al., 2012; Sanderson et al., 2013). The preliminary experiment is conducted to determine the time limited term under MCAO condition (Supplementary Figure 1; *n* = 4, *P* < 0.05). No neurological deficits were observed in the sham treated rats, whereas rats subjected to MCAO were scored 2, 3, or 4 in the neurological assessment (Supplementary Figure 5A; *n* = 20, *P* < 0.01). The neurological deficits score in GA 50 mg/kg or CsA 10 mg/kg group was significantly reduced (Supplementary Figure 5A; *n* = 20, *P* < 0.05). By immunostained with NeuN, the numbers of pyramidal neurons in the hippocampus region were diminished dramatically after reperfusion, whereas the loss of hippocampal neurons was attenuated by GA (**Figures 2F-a,b**). The expression of cleaved-caspase-3, 8 and number of cleaved-caspase-9-positive cells (% of DAPI+ cells) were dramatically increased in the MCAO group compared with sham group, suggesting that both intrinsic and extrinsic apoptosis were trigged in cerebral ischemia (**Figures 2A,C–E-a5,b5,H**; *n* = 4, *P* < 0.01). Compared with the MCAO group, pre-treatment with GA had unequally sensitive in expression of cleaved-caspase-8 (**Figures 2A,C**; *n* = 4, *P* > 0.05) and the number of cleaved-caspase-9-positive cells (% of DAPI+ cells) (**Figures 2E-b5,c5,H**; *n* = 4, *P* < 0.01), suggesting that GA alleviates neuronal apoptosis dependent in the mitochondrial apoptotic pathways.

Moreover, the mitochondrial protective effect of GA was further measured by the TEM and TTC assay. Notably, while the outer and inner membranes of mitochondria in sham group were clearly distinguishable, the mitochondria showed robustly swelling, vacuolization, disruption and loss of cristae following 48 h reperfusion (**Figures 2E-a3,b3**). This brought considerable relief in mitochondrial ultrastructure by pretreated with GA or CsA, which the cristae were slightly disrupted (**Figures 2E-c3,d3**), and the infarct volume was significantly reduced in GA pre-treatment (**Figures 2E-b1,c1,G**; *n* = 8, *P* < 0.01) further confirmed that GA effectively protects brain mitochondria against ischemia/reperfusion injury.

### GA Inhibits Cell Death and MPTP in Cultured SH-SY5Y Cells

**FIGURE 3:**
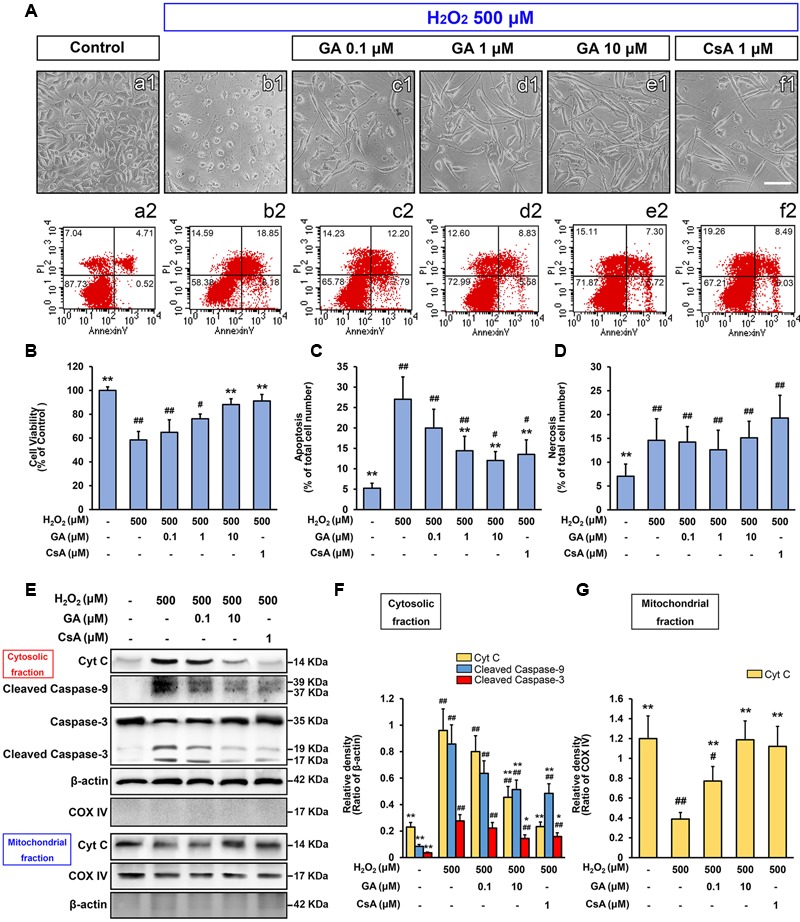
**Gallic acid inhibits H_2_O_2_-induced SH-SY5Ys death by modulating the intrinsic pathway.** The mortality and apoptosis of SH-SY5Y cells were considerably less following pretreated with GA. **(A-a2–f2)** The number of apoptosis cells were detected by flow cytometry, in accordance with the morphological observations **(A-a1–f1)**. Histogram showing the percentage of cell viability **(B)**, apoptosis **(C)** and necrosis cells **(D)**, which described as the number of positive cells/total number of cells (*n* = 5). GA inhibited the expression of mitochondrial apoptotic signaling molecules induced by H_2_O_2_. Western Blotting **(E)**, and quantification **(F,G)** the relative intensities of the bands in each sample by quantity software (*n* = 4). Scale bars, 5 μm. Data reported as the means ± SD. *P* values were obtained using two-way analysis of variance (ANOVA) test. ^##^*P* < 0.01, ^#^*P* < 0.05 versus control group. ^∗∗^*P* < 0.01, ^∗^*P* < 0.05 versus H_2_O_2_ group.

**FIGURE 4:**
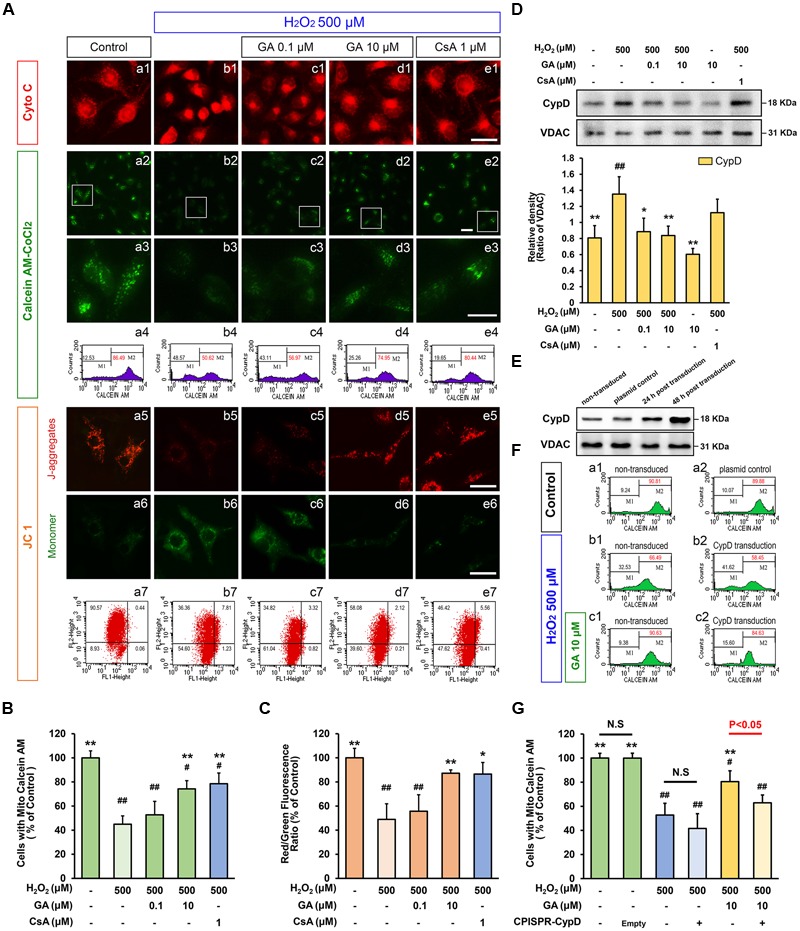
**Gallic acid desensitizes the MPTP induced by H_2_O_2_ in a CypD-dependent manner.** GA decreased the sensitivity of H_2_O_2_-induced MPTP induction in SH-SY5Y cells. **(A-a1–e1)** Representative endogenous Cyto C release in SH-SY5Y cells by Immunofluorescence analysis. **(A-a2–e2)** MPTP determined by Calcein-CoCl_2_ fluorescence. **(A-a3–e3)** Higher magnification and quantification **(A-a4–e4)** the calcein fluorescence tested by flow cytometry. The representative images show JC-1 aggregates **(A-a5–e5)**, monomers images **(A-a6–e6)**, and quantification **(A-a7–e7)** the JC-1 by flow cytometry, respectively. Histogram showing the level of calcein fluorescence **(B)**, and the ratio of JC-1 aggregates to JC-1 monomers (**C**; *n* = 4). Therapeutic effect of GA on MPTP inhibition was reversed by overexpression of CypD in SH-SY5Y cells. **(D)** Western blotting (upper) and quantification (lower) for mitochondrial CypD in SH-SY5Y cells pre-treated with GA or CsA following H_2_O_2_ for 4 h (*n* = 4). **(E)** Transfection efficiency was examined with Western Bolting. VDAC was used to loading control. **(F-a1,a2)** The levels of calcein fluorescence transfected with CypD or plasmid control, and treated with **(F-c1,c2)** or without **(F-b1,b2)** GA under H_2_O_2_ conditions were **(G)** quantificated by flow cytometry (*n* = 4). Scale bars, 5 μm. Data reported as the means ± SD. *P* values were obtained using two-way analysis of variance (ANOVA) test. N.S. indicates a *P* value > 0.05. ^##^*P* < 0.01, ^#^*P* < 0.05 versus control group. ^∗∗^*P* < 0.01, ^∗^*P* < 0.05 versus H_2_O_2_ group.

Since our *in vivo* data indicated MPTP is a key target of GA in pathologic neuron apoptosis, we next focus on validate this idea *in vitro*. Compared to the control group, H_2_O_2_ group was significantly decreased the cell viability (% of control) at 500 μM (Supplementary Figure 2; *n* = 5, *P* < 0.01), and the number of apoptotic and necrotic cells were significantly increased, in accordance with the morphological observations (**Figures 3A-a1,a2,b1,b2,C,D**; *n* = 5, *P* < 0.01). Notably, H_2_O_2_ elicited a rapid and almost complete decrease in the fluorescent density of mitochondrial calcein, a highly selective indicator of sustained MPTP opening *in situ* (Vaseva et al., 2012; Chen et al., 2015), while it was significantly reversed in the CsA pre-treated cells confirming loss of signal resulted from H_2_O_2_-induced opening of MPTP in our system (**Figures 4A-a2–a4,b2–b4,e2–e4,B**; *n* = 4, *P* < 0.01). This observation was in striking contrast to GA pre-treated cells, which considerably less SH-SY5Y cells mortality or apoptosis and retained more levels of calcein fluorescence than H_2_O_2_ treated cells in a concentration-dependent manner (**Figures 3A-b1,b2,e1,e2,B,C, 4A-b2,b4,c2,c4,d2,d4,B**; *n* = 4–5, *P* < 0.01).

As MMP could immediate dissipated by MPTP activation (Fulda et al., 2010), the level of the MMP by JC 1 assay were also examined in this work. Compared to the control group, the red/green fluorescence intensity ratio in H_2_O_2_ treatment SH-SY5Y cells was significantly decreased (**Figures 4A-a5–a7,b5–b7,C**; *n* = 4, *P* < 0.01), while GA or CsA effectively blocked this event, consistent with the results of Calcein-CoCl_2_ assay (**Figures 4A-b5–b7,d5–d7,e5–e7,C**; *n* = 4, *P* < 0.01). These data further confirmed that GA could suppress oxidative stress-induced MPTP and cell death.

### GA Inhibits SH-SY5Y Cells Death via the Mitochondrial Apoptotic Pathways

As a second line of evidence for the inhibition of GA on MPTP, the expression of mitochondrial apoptotic signaling molecules, including the initiator Cyto C, mediator caspase-9, and effector caspase-3 were measured by Western Blot. As shown in **Figure 3E**, the release of Cyto C, the expression of cleaved-caspase-9 and -3 were observed with a 4-, 9-, and 7-fold increase, respectively, when compared to the control cells (**Figures 3E,F,G,4A-a1,b1**; *n* = 4, *P* < 0.01). Notably, compared with H_2_O_2_ treated cells, GA significantly reduced the expression of Cyto C, cleaved-caspase-9 and -3 confirming that GA inhibits H_2_O_2_-induced cells death by MPTP inhibition (**Figures 3E,F,G, 4A-b1,d1**; *n* = 4, *P* < 0.05).

### GA Directly Inhibits CypD Binding to ANT-1 in Liver Mitochondria

Of note, CypD is well known bind to mitochondrial ANT, thus promoting MPTP opening (Eliseev et al., 2009; Fulda et al., 2010). Here, the physical interaction between CypD and ANT-1 was markedly enhanced by Ca^2+^ incubation, and prevented by CsA, consistent with these reports mentioned above (**Figure 1G**). GA abrogated Ca^2+^-induced CypD binding to ANT-1, indicating that the beneficial effects of GA are mediated, at least in part, by decreasing in CypD associated with ANT-1, subsequently blocking MPTP (**Figure 1G**). Moreover, the results of molecular docking between GA and CypD showed that GA was able to form electrostatic forces with ASN 102, PHE 113, MET 61, ARG 55 of CypD (**Figure 1F-c**). Notably, there is a hydrogen bond between GA and the residue ASN 102 of CypD (Distance: 2.039, Estimated free energy of binding: -0.568) (**Figure 1F-d**), in addition the binding site was correlated with previously reported binding site of CypD where CsA was bound (Kajitani et al., 2008).

### CypD Is Suppressed by GA both *In vivo* and *In vitro*

Interestingly, mitochondria isolated from mouse liver in the pre-treatment with GA once a day for 6 days (**Figure 1H**) were desensitized to the permeability transition, and the level of mitochondrial swelling triggered by Ca^2+^ or menadione was significantly decreased compared with the control mitochondria (**Figures 1I,J**; *n* = 10, *P* < 0.01). Importantly, it seemed more valence compared with mitochondria pre-treated GA 10 μM mentioned above, indicating that there may be other mechanisms at work. Western blotting data showed that not VDAC but CypD expression was significantly suppressed in liver mitochondria (**Figure 1L**; *n* = 6, *P* < 0.05), suggesting that CypD is a potential target of GA in regulating the sensitivity of MPTP in the isolated mitochondria. Moreover, no significant difference in the release of Cyto C was observed in all samples, confirmed that the mitochondrial membrane remains well (**Figure 1K**; *n* = 6, *P* > 0.05).

We reproduce this phenomenon in the cultured SH-SY5Y cells. As shown in **Figure 4D**, oxidative stress mediated by H_2_O_2_ caused greatly increase the expression level of CypD (**Figure 4D**; *n* = 4, *P* < 0.01). Compared with H_2_O_2_ treated cells, SH-SY5Y cells exposed to 0.1 or 10 μM of GA for 24 h displayed significantly decreased levels of CypD expression (**Figure 4D**; *n* = 4, *P* < 0.01). Importantly, it was worth noting that the decrease in brain mitochondrial CypD expression is indicative of the ability of GA to cross the blood-brain barrier, and suggesting that CypD is responsible for therapeutic benefit by GA via effects on MPTP inhibiting (**Figures 2A,B**; *n* = 4, *P* < 0.01).

### CypD Overexpression Abrogates GA-Induced MPTP Desensitization

To further evaluate whether CypD is essential for the sensitivity of GA in MPTP, cells were transfected with CPISPR-CypD to overexpress CypD. Incubation of SH-SY5Y cells with a CPISPR-CypD resulted in 120% increasing in CypD protein levels after 48 h transfection (**Figure 4E**; *n* = 4, *P* < 0.01). As shown in **Figure 4F**, SH-SY5Y cells overexpress CypD tended no significant loss of calcein fluorescence than non-transduced cells treated by 500 μM of H_2_O_2_ (**Figures 4F-b1,b2,G**; *n* = 4, *P* > 0.05). Importantly, the calcein fluorescence signal in the GA pretreated cells was significantly higher than H_2_O_2_ treated cells (**Figures 4F-b1,c1,G**; *n* = 4, *P* < 0.01), however, there was no significant charged in CypD overexpression SH-SY5Y cells following GA pretreated (**Figures 4F-b2,c2,G**; *n* = 4, *P* > 0.05). These results confirmed that the inhibition of H_2_O_2_-induced MPTP by GA is dependent on CypD.

### *In vitro* Modification of the ERK Phosphorylation Status Alters CypD Expression

**FIGURE 5:**
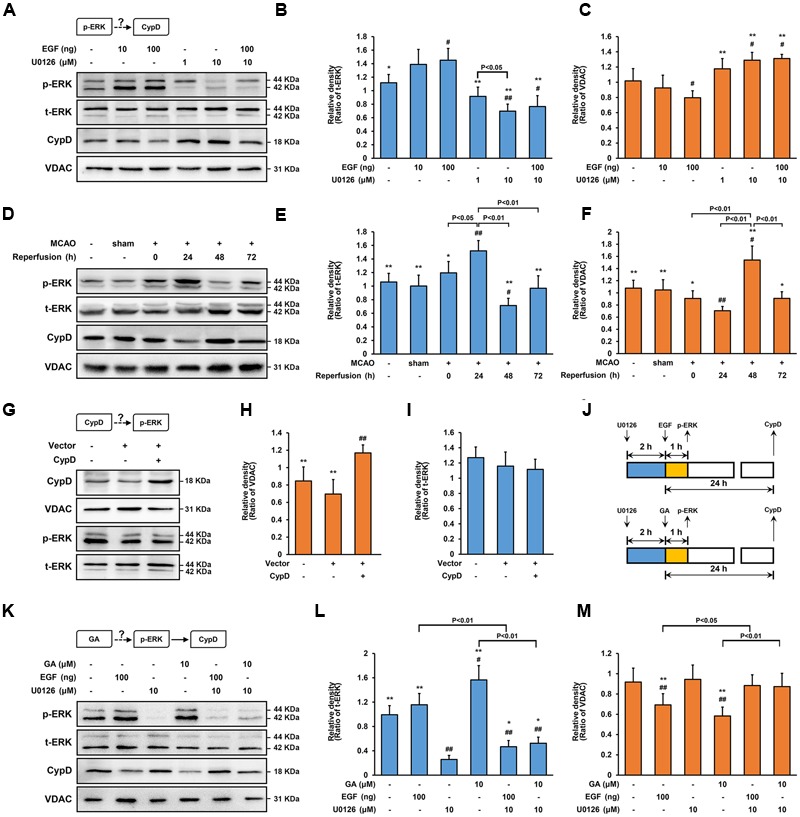
**Gallic acid alters CypD expression results in an enhancement of ERK phosphorylation.** The expression of CypD in SH-SY5Y cells partly depends on ERK phosphorylation. **(A)** the phosphorylation of ERK and the expression of CypD were determined by immunoblotting at 1 and 24 h following GA or EGF treatment, respectively. t-ERK and VDAC were used as a loading control. **(B,C)** Histogram showing the relative intensities of the bands in each sample was semi-quantification by quantity software (*n* = 4). ^##^*P* < 0.01, ^#^*P* < 0.05 versus control group. ^∗∗^*P* < 0.01, ^∗^*P* < 0.05 versus EGF (100 ng) group. **(D–F)** Transient cerebral ischemia increases ERK activity and downregulates CypD. The methods of tested and calculated the level of p-ERK and CypD were identical to (**A**; *n* = 4). ^##^*P* < 0.01, ^#^*P* < 0.05 versus sham group. ^∗∗^*P* < 0.01, ^∗^*P* < 0.05 versus Reperfusion 24 h group. CypD was a downstream target protein of ERK. **(G)** Transfection efficiency was examined with Western bolt. **(H,I)** The phosphorylation of ERK and the expression of CypD were determined by immunoblotting (*n* = 4). **(J)** The design for the *in vitro* experiment in **(K–M)** GA altered CypD expression through potentiating ERK phosphorylation. The methods of tested and calculated the level of p-ERK and CypD were identical to (**A**; *n* = 4).^##^*P* < 0.01, ^#^*P* < 0.05 versus control group. ^∗∗^*P* < 0.01, ^∗^*P* < 0.05 versus U0126 (10 μM) group. Data reported as the means ± SD. *P* values were obtained using two-way analysis of variance (ANOVA) test.

Of note, ERK is involved in mitochondrial dysfunction during both apoptotic and autophagy cell death-inducing pathways (Monick et al., 2008; Martinez-Lopez et al., 2013). As showed in **Figure 5**, EGF activated ERK in a concentration dependent manner, and it exerted the best effect at a concentration of 100 ng for 1 h (**Figures 5A,B**; *n* = 4, *P* < 0.05). As expected, the level of phosphorylation ERK (p-ERK) were significantly inhibited by pre-treatment with U0126, a specificity inhibitor of ERK phosphorylation, at a concentration of 10 μM (**Figures 5A,B**; *n* = 4, *P* < 0.01). In comparison with control cells, U0126 strongly increased the expression of CypD following 24 h of exposure, while EGF has the exactly opposite effect to that of CypD expression (**Figures 5A,C**; *n* = 4, *P* < 0.05). Importantly, U0126 reversed the EGF-induced CypD overexpression, suggesting that the CypD expression in SH-SY5Y cells, at least in part, depends on ERK phosphorylation (**Figures 5A–C**; *n* = 4, *P* < 0.05).

Additionally, to explore whether CypD also regulated the phosphorylation of ERK, SH-SY5Y cells were transfected CPISPR-CypD to overexpress CypD. Incubation of SH-SY5Y cells with a CPISPR-CypD resulted in 105% increasing in CypD protein levels after 48 h transfection (**Figures 5G,H**; *n* = 4, *P* < 0.01). Compared with the control cells, CypD overexpression had no effect on the level of p-ERK, suggesting that CypD was a downstream target protein of ERK (**Figures 5G,I**; *n* = 4, *P* > 0.05).

### Alteration of CypD Expression by ERK Phosphorylation in MCAO Rats

As a second line of evidence for the signaling of ERK-CypD, the effect of ERK in CypD expression during cerebral ischemia/reperfusion injury were studied *in vivo*. Of note, robust induction of p-ERK was expressed in the ischemic penumbra at the early stage of reperfusion, which involved in neuronal loss through caspase-3-mediated apoptosis (Li et al., 2002; Liang et al., 2014). In comparison with sham rats, the level of p-ERK were significantly increased at 24 h, strongly declined at 48 h, and recovered at 72 h in the ischemic penumbral region following cerebral ischemia/reperfusion injury (**Figures 5D,E**; *n* = 4, *P* < 0.01). Similarly, corresponds to overexpression of p-ERK, the expression of CypD were significantly decreased at 24 h, while robustly increased at 48 h after cerebral ischemia/reperfusion injury (**Figures 5D,F**; *n* = 4, *P* < 0.05). Importantly, compared with sham group, the cleaved-caspase-3 were dramatically increased at 48 h following cerebral ischemia/reperfusion injury (Supplementary Figure 1; *n* = 4, *P* < 0.05), further indicating that CypD-ERK axis involved in neuronal apoptosis at the early stage of reperfusion.

### ERK Mediates GA-Induced Cytoprotective Effect via CypD Downregulation

**FIGURE 6:**
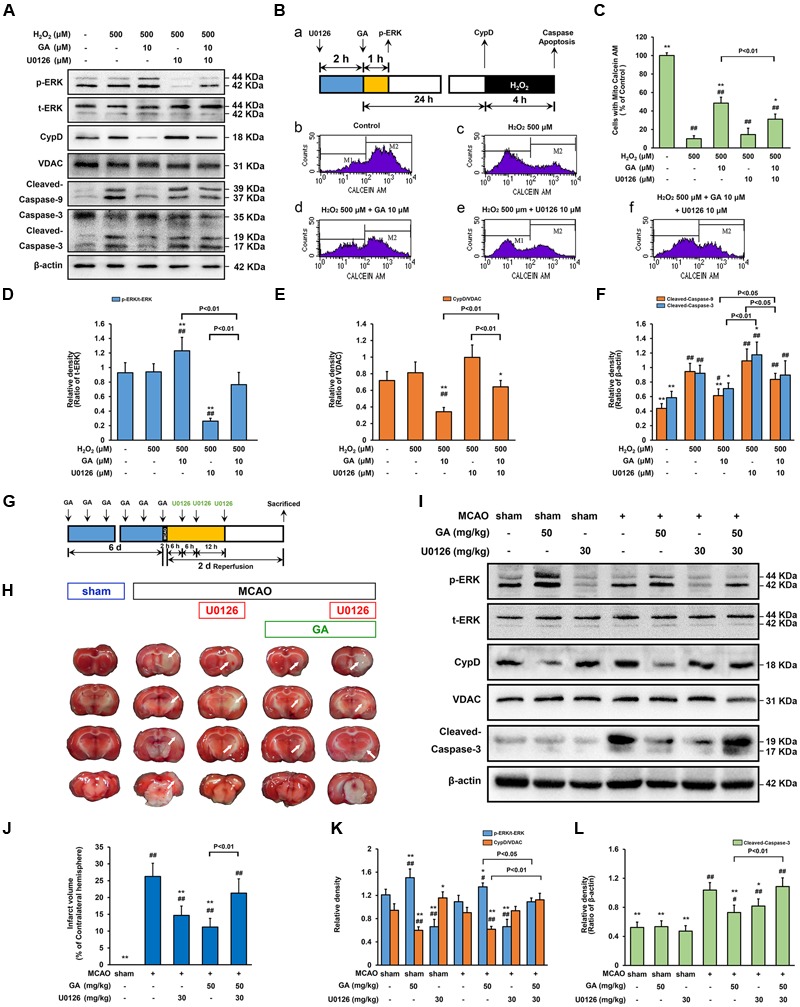
**Extracellular signal-regulated kinases mediates GA-induced cytoprotective effect via CypD downregulation.** ERK is necessary for GA to prevent H_2_O_2_-induced apoptosis via CypD downregulation. **(A)** The phosphorylation of ERK and the expression of CypD were determined by immunoblotting. t-ERK, VDAC, and β-actin were used as a loading control. The design for the *in vitro* experiment in this figure was shown in **(B-a)**. **(B-b–f)** MPTP determined by Calcein-CoCl_2_ assay and **(C)** quantification by flow cytometry (*n* = 4). **(D–F)** Histogram showing the relative intensities of the bands in each sample was semi-quantification by quantity software (*n* = 4). ^##^*P* < 0.01, ^#^*P* < 0.05 versus control group. ^∗∗^*P* < 0.01, ^∗^*P* < 0.05 versus H_2_O_2_ group. ERK signaling triggered by GA were involved in MCAO-mediated apoptosis in rats. **(G)** The design for the *in vivo* experiment in this figure. **(H)** Representative TTC-stained coronal brain sections; white indicates infarcted tissue. **(I)** The phosphorylation of ERK and the expression of CypD were determined by immunoblotting. **(J)** Histogram showing the infarct volume (% of contralateral hemisphere) in TTC-stained brain sections (*n* = 8). **(K,L)** Quantification the bands in each sample was semi-quantification by quantity software (*n* = 4). ^##^*P* < 0.01, ^#^*P* < 0.05 versus sham group. ^∗∗^*P* < 0.01, ^∗^*P* < 0.05 versus MCAO group. Data reported as the means ± SD. *P* values were obtained using two-way analysis of variance (ANOVA) test.

Since our data identified that phosphorylation of ERK is an early signaling event before the expression of CypD, whether ERK signaling participate in GA-induced CypD expression was determine. In SH-SY5Y cells, the phosphorylation of ERK is elevated in cells exposed to GA for 1 h when compared with control group (**Figures 5K,L, 6A,D**; *n* = 4, *P* < 0.05). Notably, the GA-induced downregulation of CypD was abrogated by U0126, suggesting that the effect of GA on CypD, at least in part, results in an enhancement of ERK phosphorylation (**Figures 5K–M, 6A,E**; *n* = 4, *P* < 0.01). The neurological deficits score in GA 50 mg/kg or U0126 30 mg/kg group was significantly reduced (Supplementary Figure 5B; *n* = 12, *P* > 0.05). Moreover, while U0126 significantly reduced the percent of the infarct volume and the expression of cleaved-caspase-3 following MCAO insult *in vivo* (**Figures 6H–J,L**; *n* = 4–8, *P* < 0.05), no significant difference in the level of calcein fluorescence between U0126 and H_2_O_2_ group *in vitro* (**Figures 6B-c,d,e,C**; *n* = 4, *P* > 0.05).

While GA was able to desensitize the MPTP in a CypD-dependent manner, whether this protection effect was related to ERK phosphorylation was investigated. As shown in **Figure 6**, combination treatment with U0126 abrogated GA-induced CypD downregulation, MPTP inhibition, and promoted caspase activation *in vitro* (**Figures 6A,B-c,d,f,C,F**; *n* = 4, *P* > 0.05) and *in vivo* (**Figures 6H–L**; *n* = 4, *P* > 0.05), further confirmed the idea that ERK mediates GA-induced cytoprotective effect via CypD downregulation.

## Discussion

Here, for the first time, it demonstrates that the natural GA is a novel small molecule inhibitor of MPTP, which established this neuroprotective effect depending on regulating the ERK-CypD axis. Moreover, they are in contradiction with previous reports that inhibiting ERK phosphorylation could significantly attenuate apoptosis following cerebral ischemia/reperfusion injury (Wang et al., 2003; Wang T. et al., 2015).

Mitochondrial permeability transition pore results from relatively severe perturbations of intracellular redox and/or Ca^2+^ homeostasis, such as those that can be artificially imposed by ionophores (e.g., CaCl_2_) or potent oxidants (e.g., H_2_O_2_, menadione) *in vitro* (Vaseva et al., 2012; Chen et al., 2015), or those that are caused by ischemia/reperfusion insults *in vivo* (Schinzel et al., 2005; Sun et al., 2012). Earlier studies by our group identified that GA is a potential mitochondrial protective agent, which has significant neuroprotective effect in cerebral ischemia/reperfusion injury (Sun et al., 2014). Here, pretreatment with GA has a higher Ca^2+^ threshold, more refractory to mitochondrial swelling, calcein fluorescence and MMP dissipated induced by potent oxidants, deepen the knowledge on molecular actions triggered by GA, which result in MPTP inhibition. GA significantly reduced the expression of mitochondrial apoptotic signaling molecules further verify the above results. This similar results were also observed *in vivo*. Of note, cerebral ischemia triggers both intrinsic pathway that originates from mitochondrial crash associated stimulation of caspase-9 and the extrinsic pathway that derives from death receptors activation and subsequent stimulation of caspase-8 (Broughton et al., 2009). GA brought considerable relief in brain mitochondrial ultrastructure, and had unequally sensitive in caspase-8 and 9 activation, emphasizing that GA alleviates neuronal apoptosis dependent in the mitochondrial apoptotic pathways. Totally, these *in vivo* and *in vitro* data demonstrated that GA is a genuine inhibitor of MPTP.

Indeed, cells depleted of putative core components of MPTP or treated with pharmacological modulators thereof exhibit an altered sensitivity to MPTP induction. However, whereas the generation of *Ppif^-/-^* mice and mice pretreatment with CsA formally attributed to CypD a key function in the molecular cascades that precipitate MPTP (Baines et al., 2005; Schinzel et al., 2005; Hu et al., 2010; Vaseva et al., 2012; Cho et al., 2013), the simultaneous knockout of Slc25a4/5 (encoding ANT-1/2) or VDAC-1/2/3 failed to protect specific cells from MPTP induction in mouse (Kokoszka et al., 2004; Baines et al., 2007). At present, not VDAC but CypD were in significant depletion of GA affected cells both *in vivo* and *in vitro*, which abolished by CypD overexpression confirming GA desensitizes the MPTP induced by H_2_O_2_ is CypD dependent. This may explain why pretreated with GA was equally well protected SH-SY5Y cells from MPTP induction induced by H_2_O_2_ as CsA group, while GA has lower potency as a CypD inhibitor when compared with CsA. Thus, our findings confirmed that GA modulated the sensitivity of the MPTP through direct and indirect-interference with CypD.

The quest for key upstream regulators of the CypD yielded no better results. Actually, the transient or stable knockdown of p53, glycogen synthase kinase 3β, as well as F_1_F_O_ ATP-synthase, has been shown to modulate the sensitivity of cultured cells to MPTP-inducing stimuli by binding to CypD (Nishihara et al., 2007; Vaseva et al., 2012; Elrod and Molkentin, 2013). In particular, only a few of these proteins have been mechanistically implicated in MPTP *in vivo* so far. Here, ERK has been etiologically implicated in brain injury following cerebral ischemia/reperfusion owing to its ability to inhibit MPTP by downregulating CypD expression, and the resistance displayed by GA were abrogated by co-administration of U0126 *in vitro* and *in vivo*, further confirming that the neuroprotective effect of GA is depending on regulating the ERK-CypD axis.

Of note, robust induction of ERK was expressed in the ischemic penumbra at the early stage of reperfusion, which involved in neuronal loss through caspase-3-mediated apoptosis (Li et al., 2002; Liang et al., 2014). At present, the cleaved-caspase-3 were dramatically increased at 48 h following reperfusion, while the expression of ERK were decreased, which provides a direct approach in understanding the phosphorylation of ERK is beneficial to the recovery of neurons after cerebral ischemia. Our results challenge the view that inhibiting ERK activation could prevent neuronal damage following cerebral ischemia/reperfusion injury (Wang et al., 2003; Wang T. et al., 2015). At present, while U0126 has significant neuroprotective effect in cerebral ischemia/reperfusion injury, it did not promote cell survival following H_2_O_2_ injury. Of note, CypD-regulated MPTP is a crucial event during H_2_O_2_-induced cell death, definitively established by the CypD knockout mice (Baines et al., 2005; Vaseva et al., 2012). Hence, the failure of U0126 for H_2_O_2_-induced cell death could be understand to their inability to modulate MPTP. Importantly, ERK phosphorylation-induced apoptosis is activated in a Ca^2+^/Calmodulin (CaM)-dependent manner following cerebral ischemia (Zhao et al., 2008). Despite GA probably not only targeted ERK-CypD pathway, the successful of GA as ERK activator for experimental stroke may be due to their ability to direct modulate mitochondrial CRC via MPTP inhibition.

Additionally, though GA has significant neuroprotective effects by inhibiting MPTP, the influence on the regulation of CYP450 by GA could not be ignored. Several studies have revealed that GA weakly and time-dependently inactivated CYP3A4 via its oxidative products, leading to more potential for toxicity of co-administered drugs (Stupans et al., 2002; Pu et al., 2015; Vijayakumar et al., 2015). Of note, CYP3A4 plays a key role in metabolic clearance of Nifedipine and Amlodipine in humans, which were commonly prescribed calcium channel blocker for the treatment of hypertension (Zhu et al., 2014; Wang X.F. et al., 2015). As a major cardiovascular risk factor for stroke, many patients of ischemic stroke are taking hypertensive medications (Bartsch et al., 2013). Blind applying GA in stroke patients suffered from hypertension will be blocked the metabolism of calcium antagonists, and may cause adverse reactions. Therefore, detailed studies on the interaction of GA and cardiovascular drugs have important practical significance, in which, the regulating of CYP450 systems are often required.

## Conclusion

**FIGURE 7:**
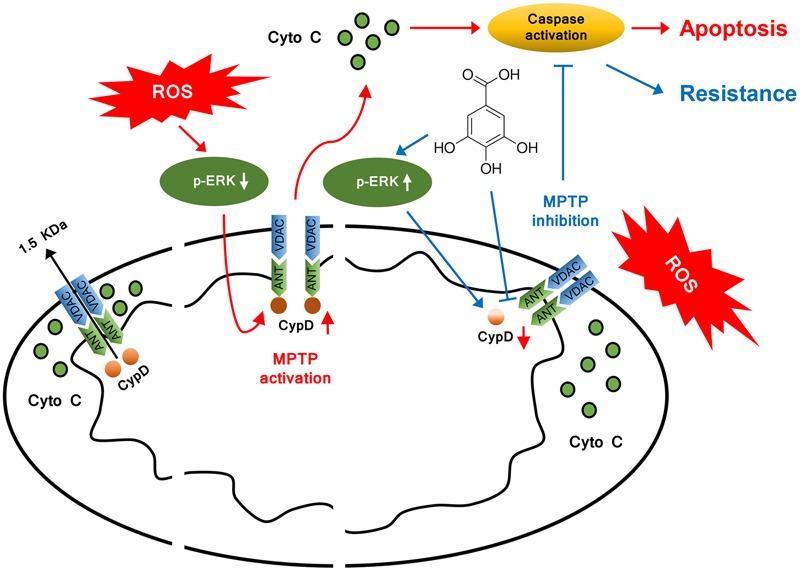
**Model illustrating the mechanism of cytoprotective effect by GA.** In addition to inhibit CypD binding to ANT, GA potentiates ERK phosphorylation, leading to a decrease in CypD expression, resulting in a desensitization to induction of MPTP, thus inhibiting caspase activation and ultimately giving rise to cellular survival.

This study identifies GA is a novel MPTP inhibitor, which might be promising for the treatment of stroke. We confirms CypD as a target protein through which GA exposure suppress the sensitivity of mitochondria to induction of MPTP, and proposes a model explaining the possible mechanism behind the downregulation CypD by GA in **Figure 7**. In consideration that MPTP plays a crucial role in mediating mitochondrial dysfunction cause for oxidative stress, we believe that GA could be beneficial in the treatment of wide variety of diseases (Sun et al., 2012; Andreux et al., 2013; Sanderson et al., 2013).

## Author Contributions

JS designed the study, coordinated with different investigators, and supervised the whole group. JS, D-DR, and CC performed majority of experiments; J-YW, DC, and HY analyzed data. C-LF provided data for molecular docking and participated in discussing. JS, J-YW, and JG wrote and generated final draft of the manuscript.

## Conflict of Interest Statement

The authors declare that the research was conducted in the absence of any commercial or financial relationships that could be construed as a potential conflict of interest.

## Abbreviations

ANT: adenine nucleotide translocator
CPISPR: clustered regularly interspaced short palindromic repeats
CRC: calcium retention capacity
CsA: cyclosporin A
CYP450: cytochrome P450
CypD: cyclophilin D
Cyto C: cytochrome C
DAPI: 4′,6-diamidino-2-phenylindole
EGF: epidermal growth factor
ERK: extracellular signal-regulated kinase
FITC: fluorescein isothiocyanate
GA: gallic acid
H & E: haematoxylin and eosin
JC-1: 5,5′,6,6′-tetrachloro-1,1′,3,3′-tetraethylbenzimidazol carbocyanine iodide
MCAO: middle cerebral artery occlusion
MMP: mitochondrial membrane potential
MPTP: mitochondrial permeability transition pore
MTT: 3-(4,5-dimethylthiazol-2-yl)-2,5-diphenyltetrazolium bromide
NeuN: neuronal nuclei
PI: propidium iodide
rCBF: regional cerebral blood flow
ROS: reactive oxidative species
TEM: transmission electron microscopy
TTC: 2,3,5-triphenyl-tetrazolium chloride
U0126: 1,4-diamino-2,3-dicyano-1,4-bis [2-aminophenylthio] butadiene
VDAC: voltage-dependent anion channel

## References

[B1] Andreux P. A. Houtkooper R. H. Auwerx J. (2013). Pharmacological approaches to restore mitochondrial function. *Nat. Rev. Drug Discov.* 12 465–483. 10.1038/nrd4023 23666487 PMC3896945

[B2] Baines C. P. Kaiser R. A. Purcell N. H. Blair N. S. Osinska H. Hambleton M. A. (2005). Loss of cyclophilin D reveals a critical role for mitochondrial permeability transition in cell death. *Nature* 434 658–662. 10.1038/nature03434 15800627

[B3] Baines C. P. Kaiser R. A. Sheiko T. Craigen W. J. Molkentin J. D. (2007). Voltage-dependent anion channels are dispensable for mitochondrial-dependent cell death. *Nat. Cell Biol.* 9 550–555. 10.1038/ncb1575 17417626 PMC2680246

[B4] Bartsch J. A. Teare G. F. Neufeld A. Hudema N. Muhajarine N. (2013). Secondary prevention of stroke in Saskatchewan, Canada: hypertension control. *Int. J. Stroke* 8(Suppl. A100) 32–38. 10.1111/j.1747-4949.2012.00930.x 23088414

[B5] Baumgartner H. K. Gerasimenko J. V. Thorne C. Ferdek P. Pozzan T. Tepikin A. V. (2009). Calcium elevation in mitochondria is the main Ca2+ requirement for mitochondrial permeability transition pore (mPTP) opening. *J. Biol. Chem.* 284 20796–20803. 10.1074/jbc.M109.025353 19515844 PMC2742844

[B6] Bonora M. Morganti C. Morciano G. Giorgi C. Wieckowski M. R. Pinton P. (2016). Comprehensive analysis of mitochondrial permeability transition pore activity in living cells using fluorescence-imaging-based techniques. *Nat. Protoc.* 11 1067–1080. 10.1038/nprot.2016.064 27172167

[B7] Bonora M. Pinton P. (2014). The mitochondrial permeability transition pore and cancer: molecular mechanisms involved in cell death. *Front. Oncol.* 4:302 10.3389/fonc.2014.00302 PMC4235083 25478322

[B8] Bonora M. Wieckowsk M. R. Chinopoulos C. Kepp O. Kroemer G. Galluzzi L. (2015). Molecular mechanisms of cell death: central implication of ATP synthase in mitochondrial permeability transition. *Oncogene* 34 1475–1486. 10.1038/onc.2014.462 24727893

[B9] Broughton B. R. Reutens D. C. Sobey C. G. (2009). Apoptotic mechanisms after cerebral ischemia. *Stroke* 40 e331–e339. 10.1161/STROKEAHA.108.531632 19182083

[B10] Chen P. Hu Y. F. Wang L. Xiao W. F. Bao X. Y. Pan C. (2015). Mitochondrial apoptotic pathway is activated by H2O2-mediated oxidative stress in BmN-SWU1 cells from *Bombyx mori* ovary. *PLoS ONE* 10:e0134694 10.1371/journal.pone.0134694 PMC4520666 26225758

[B11] Cho T. H. Aguettaz P. Campuzano O. Charriaut-Marlangue C. Riou A. Berthezène Y. (2013). Pre- and post-treatment with cyclosporine A in a rat model of transient focal cerebral ischaemia with multimodal MRI screening. *Int. J. Stroke* 8 669–674. 10.1111/j.1747-4949.2012.00849.x 22882746

[B12] Criddle D. N. Gillies S. Baumgartner-Wilson H. K. Jaffar M. Chinje E. C. Passmore S. (2006). Menadione-induced reactive oxygen species generation via redox cycling promotes apoptosis of murine pancreatic acinar cells. *J. Biol. Chem.* 281 40485–40492. 10.1074/jbc.M607704200 17088248

[B13] Eliseev R. A. Malecki J. Lester T. Zhang Y. Humphrey J. Gunter T. E. (2009). Cyclophilin D interacts with Bcl 2 and exerts an anti-apoptotic effect. *J. Biol. Chem.* 284 9692–9699. 10.1074/jbc.M808750200 19228691 PMC2665090

[B14] Elrod J. W. Molkentin J. D. (2013). Physiologic functions of cyclophilin D and the mitochondrial permeability transition pore. *Circ. J.* 77 1111–1122. 10.1253/circj.CJ-13-0321 23538482 PMC6397958

[B15] Fernandez-Godino R. Garland D. L. Pierce E. A. (2016). Isolation, culture and characterization of primary mouse RPE cells. *Nat. Protoc.* 11 1206–1218. 10.1038/nprot.2016.065 27281648 PMC6432639

[B16] Flynn R. W. MacWalter R. S. Doney A. S. (2008). The cost of cerebral ischemia. *Neuropharmacology* 55 250–256. 10.1016/j.neuropharm.2008.05.031 18573263

[B17] Fulda S. Galluzzi L. Kroemer G. (2010). Targeting mitochondria for cancer therapy. *Nat. Rev. Drug. Discov.* 9 447–464. 10.1038/nrd3137 20467424

[B18] Galat A. Metcalfe S. M. (1995). Peptidylproline cis/trans isomerases. *Prog. Biophys. Mol. Biol.* 63 67–118. 10.1016/0079-6107(94)00009-X 7538221

[B19] Ganot N. Meker S. Reytman L. Tzubery A. Tshuva E. Y. (2013). Anticancer metal complexes: synthesis and cytotoxicity evaluation by the MTT assay. *J. Vis. Exp.* e50767 10.3791/50767 PMC3989495 24300943

[B20] Hu W. Chen Z. Ye Z. Xia D. Xia Z. Ma J. (2010). Knockdown of cyclophilin D gene by RNAi protects rat from ischemia/reperfusion-induced renal injury. *Kidney Blood Press. Res.* 33 193–199. 10.1159/000316704 20588055

[B21] Kajitani K. Fujihashi M. Kobayashi Y. Shimizu S. Tsujimoto Y. Miki K. (2008). Crystal structure of human cyclophilin D in complex with its inhibitor, cyclosporin A at 0.96-A resolution. *Proteins* 70 1635–1639. 10.1002/prot.21855 18076075

[B22] Kokoszka J. E. Waymire K. G. Levy S. E. Sligh J. E. Cai J. Jones D. P. (2004). The ADP/ATP translocator is not essential for the mitochondrial permeability transition pore. *Nature* 427 461–465. 10.1038/nature02229 14749836 PMC3049806

[B23] Kroemer G. Galluzzi L. Brenner C. (2007). Mitochondrial membrane permeabilization in cell death. *Physiol. Rev.* 87 99–163. 10.1152/physrev.00013.2006 17237344

[B24] Leker R. R. Gai N. Mechoulam R. Ovadia H. (2003). Drug-induced hypothermia reduces ischemic damage: effects of the cannabinoid HU-210. *Stroke* 34 2000–2006. 10.1161/01.STR.0000079817.68944.1E 12829867

[B25] Li F. Omori N. Sato K. Jin G. Nagano I. Manabe Y. (2002). Coordinate expression of survival p-ERK and proapoptotic cytochrome c signals in rat brain neurons after transient MCAO. *Brain Res.* 958 83–88. 10.1016/S0006-8993(02)03465-0 12468032

[B26] Liang X. Hu Q. Li B. McBride D. Bian H. Spagnoli P. (2014). Follistatin-like 1 attenuates apoptosis via disco-interacting protein 2 homolog A/Akt pathway after middle cerebral artery occlusion in rats. *Stroke* 45 3048–3054. 10.1161/STROKEAHA.114.006092 25139876 PMC4174959

[B27] Longa E. Z. Weinstein P. R. Carlson S. Cummins R. (1989). Reversible middle cerebral artery occlusion without craniectomy in rats. *Stroke* 20 84–91. 10.1161/01.STR.20.1.84 2643202

[B28] Maddahi A. Edvinsson L. (2008). Enhanced expressions of microvascular smooth muscle receptors after focal cerebral ischemia occur via the MAPK MEK/ERK pathway. *BMC Neurosci.* 9:85 10.1186/1471-2202-9-85 PMC2553085 18793415

[B29] Mao X. W. Pan C. S. Huang P. Liu Y. Y. Wang C. S. Yan L. (2015). Levo-tetrahydropalmatine attenuates mouse blood-brain barrier injury induced by focal cerebral ischemia and reperfusion: involvement of Src kinase. *Sci. Rep.* 5:11155 10.1038/srep11155 PMC4461916 26059793

[B30] Martin L. J. Fancelli D. Wong M. Niedzwiecki M. Ballarini M. Plyte S. (2014). GNX-4728, a novel small molecule drug inhibitor of mitochondrial permeability transition, is therapeutic in a mouse model of amyotrophic lateral sclerosis. *Front. Cell Neurosci.* 8:433 10.3389/fncel.2014.00433 PMC4271619 25565966

[B31] Martinez-Lopez N. Athonvarangkul D. Mishall P. Sahu S. Singh R. (2013). Autophagy proteins regulate ERK phosphorylation. *Nat. Commun.* 4:2799 10.1038/ncomms3799 PMC3868163 24240988

[B32] Monaghan M. G. Kroll S. Brucker S. Y. Schenke-Layland K. (2016). Enabling multiphoton and second harmonic generation imaging in paraffin-embedded and histologically stained sections. *Tissue Eng. Part C Methods* 22 517–523. 10.1089/ten.TEC.2016.0071 27018844 PMC4922008

[B33] Monick M. M. Powers L. S. Barrett C. W. Hinde S. Ashare A. Groskreutz D. J. (2008). Constitutive ERK MAPK activity regulates macrophage ATP production and mitochondrial integrity. *J. Immunol.* 180 7485–7496. 10.4049/jimmunol.180.11.7485 18490749 PMC2410094

[B34] Muramatsu Y. Furuichi Y. Tojo N. Moriguchi A. Maemoto T. Nakada H. (2007). Neuroprotective efficacy of FR901459, a novel derivative of cyclosporin A, in in vitro mitochondrial damage and in vivo transient cerebral ischemia models. *Brain Res.* 1149 181–190. 10.1016/j.brainres.2007.02.036 17391653

[B35] Nishihara M. Miura T. Miki T. Tanno M. Yano T. Naitoh K. (2007). Modulation of the mitochondrial permeability transition pore complex in GSK-3beta-mediated myocardial protection. *J. Mol. Cell Cardiol.* 43 564–570. 10.1016/j.yjmcc.2007.08.010 17931653

[B36] Pal C. Bindu S. Dey S. Alam A. Goyal M. Iqbal M. S. (2010). Gallic acid prevents nonsteroidal anti-inflammatory drug-induced gastropathy in rat by blocking oxidative stress and apoptosis. *Free Radic. Biol. Med.* 49 258–267. 10.1016/j.freeradbiomed.2010.04.013 20406680

[B37] Pu Q. H. Shi L. Yu C. (2015). Time-dependent inhibition of CYP3A4 by gallic acid in human liver microsomes and recombinant systems. *Xenobiotica* 45 213–217. 10.3109/00498254.2014.973470 25322914

[B38] Rezzani R. (2004). Cyclosporine A and adverse effects on organs: histochemical studies. *Prog. Histochem. Cytochem.* 39 85–128. 10.1016/j.proghi.2004.04.001 15354618

[B39] Sanderson T. H. Reynolds C. A. Kumar R. Przyklenk K. Hüttemann M. (2013). Molecular mechanisms of ischemia-reperfusion injury in brain: pivotal role of the mitochondrial membrane potential in reactive oxygen species generation. *Mol. Neurobiol.* 47 9–23. 10.1007/s12035-012-8344-z 23011809 PMC3725766

[B40] Schinzel A. C. Takeuchi O. Huang Z. Fisher J. K. Zhou Z. Rubens J. (2005). Cyclophilin D is a component of mitochondrial permeability transition and mediates neuronal cell death after focal cerebral ischemia. *Proc. Natl. Acad. Sci. U.S.A.* 102 12005–12010. 10.1073/pnas.0505294102 16103352 PMC1189333

[B41] Shen Y. Yang H. Xia G. Wang J. Cai B. Jia X. (2013). Isolation of gallic acid and methyl gallate from folium Toonea sinensis and validated method for their quantitation using LC-based technologies. *Acta. Chromatogr.* 25 687–701. 10.1556/AChrom.25.2013.4.7

[B42] Sims N. R. Anderson M. F. (2002). Mitochondrial contributions to tissue damage in stroke. *Neurochem. Int.* 40 511–526. 10.1016/S0197-0186(01)00122-X 11850108

[B43] Stupans L. Tan H. W. Kirlich A. Tuck K. Hayball P. Murray M. (2002). Inhibition of CYP3A-mediated oxidation in human hepatic microsomes by the dietary derived complex phenol, gallic acid. *J. Pharm. Pharmacol.* 54 269–275. 10.1211/0022357021778303 11848291

[B44] Sun J. Li Y. Z. Ding Y. H. Wang J. Geng J. Yang H. (2014). Neuroprotective effects of gallic acid against hypoxia/reoxygenation-induced mitochondrial dysfunctions in vitro and cerebral ischemia/reperfusion injury in vivo. *Brain Res.* 1589 126–139. 10.1016/j.brainres.2014.09.039 25251593

[B45] Sun J. Luan Q. Dong H. Song W. Xie K. Hou L. (2012). Inhibition of mitochondrial permeability transition pore opening contributes to the neuroprotective effects of ischemic postconditioning in rats. *Brain Res.* 1436 101–110. 10.1016/j.brainres.2011.11.055 22197697

[B46] Timiri Shanmugam P. S. Nair R. P. De Benedetti A. Caldito G. Abreo F. Sunavala-Dossabhoy G. (2016). Tousled kinase activator, gallic acid, promotes homologous recombinational repair and suppresses radiation cytotoxicity in salivary gland cells. *Free Radic. Biol. Med.* 93 217–226. 10.1016/j.freeradbiomed.2015.12.029 26855419 PMC5257199

[B47] Vanden Berghe T. Linkermann A. Jouan-Lanhouet S. Walczak H. Vandenabeele P. (2014). Regulated necrosis: the expanding network of non-apoptotic cell death pathways. *Nat. Rev. Mol. Cell Biol.* 15 135–147. 10.1038/nrm3737 24452471

[B48] Vaseva A. V. Marchenko N. D. Ji K. Tsirka S. E. Holzmann S. Moll U. M. (2012). p53 opens the mitochondrial permeability transition pore to trigger necrosis. *Cell* 149 1536–1548. 10.1016/j.cell.2012.05.014 22726440 PMC3383624

[B49] Vijayakumar T. M. Kumar R. M. Agrawal A. Dubey G. P. Ilango K. (2015). Comparative inhibitory potential of selected dietary bioactive polyphenols, phytosterols on CYP3A4 and CYP2D6 with fluorometric high-throughput screening. *J. Food Sci. Technol.* 52 4537–4543. 10.1007/s13197-014-1472-x 26139922 PMC4486538

[B50] Wang T. Zhai L. Zhang H. Zhao L. Guo Y. (2015). Picroside II inhibits the MEK-ERK 1/2-COX 2 signal pathway to prevent cerebral ischemic injury in rats. *J. Mol. Neurosci.* 57 335–351. 10.1007/s12031-015-0623-5 26240040

[B51] Wang X. Wang H. Xu L. Rozanski D. J. Sugawara T. Chan P. H. (2003). Significant neuroprotection against ischemic brain injury by inhibition of the MEK1 protein kinase in mice: exploration of potential mechanism associated with apoptosis. *J. Pharmacol. Exp. Ther.* 304 172–178. 10.1124/jpet.102.040246 12490588

[B52] Wang X. F. Yan L. Cao H. M. Wei L. M. Yang W. H. Zhang S. J. (2015). Effect of CYP3A4^∗^1G, CYP3A5^∗^3, POR^∗^28, and ABCB1 C3435T on the pharmacokinetics of nifedipine in healthy Chinese volunteers. *Int. J. Clin. Pharmacol. Ther.* 53 737–745. 10.5414/CP202211 26104034

[B53] Zhang T. Zhang Y. Cui M. Jin L. Wang Y. Lv F. (2016). CaMKII is a RIP 3 substrate mediating ischemia-and oxidative stress-induced myocardial necroptosis. *Nat. Med.* 22 175–182. 10.1038/nm.4017 26726877

[B54] Zhao J. Wu H. W. Chen Y. J. Tian H. P. Li L. X. Han X. (2008). Protein phosphatase 2A-negative regulation of the protective signaling pathway of Ca2+/CaM-dependent ERK activation in cerebral ischemia. *J. Neurosci. Res.* 86 2733–2745. 10.1002/jnr.21712 18478546

[B55] Zhu Y. Wang F. Li Q. Zhu M. Du A. Tang W. (2014). Amlodipine metabolism in human liver microsomes and roles of CYP3A4/5 in the dihydropyridine dehydrogenation. *Drug Metab. Dispos.* 42 245–249. 10.1124/dmd.113.055400 24301608

