# Supplementary material for: Desensitizing Mitochondrial Permeability Transition by ERK-Cyclophilin D Axis Contributes to the Neuroprotective Effect of Gallic Acid against Cerebral Ischemia/Reperfusion Injury
Source: Front Pharmacol. 2017 Apr 6;8:184. doi: 10.3389/fphar.2017.00184 (PMC5382198; doi:10.3389/fphar.2017.00184)
Supplement: Supplementary file 1 [file Data_Sheet_1.PDF]

Supplementary Material

Desensitizing mitochondrial permeability transition by ERK-cyclophilin D axis contributes to the neuroprotective effect of gallic acid against cerebral ischemia/reperfusion injury

Jing Sun [1, 2]*, Da-Dui Ren [1], Jin-Yi Wan [2], Chen Chen [1], Dong Chen [1], Huan Yang [2], Chun-Lai Feng [3], Jing Gao [1]*

*** Correspondence:**

Corresponding Author: Jing Sun & Jing Gao

Neurobiology & Mitochondrial Laboratory,

School of Pharmacy,

Jiangsu University,

NO.301 Xuefu Road, Zhenjiang 212013, P.R. China.

Tel.: 86-511-88791552

Fax: 86-511-85038451-806

Email: sjyancheng@aliyun.com; jinggao@ujs.edu.cn

1. **Supplementary Material**

1.1 Chemicals and antibodies

GA and CsA were purchased from Cayman Chemical (Shanghai, China); H_2O_2, $CoCl_2$, menadione sodium bisulfite, MTT, and TTC were purchased from Sigma Aldrich (Beijing, China); U0126, anti-Cyto C, COX IV, caspase-3, 8, Cleaved-caspase-9, ERK 1/2, and Phospho-ERK 1/2 antibody were purchased from Cell Signaling Technology (Shanghai, China); Anti-CypD and Neuronal nuclei (NeuN) antibody were purchased from Millipore (Shanghai, China); Anti-VDAC and β-actin antibody were purchased from Abcam (Beijing, China); Calcein-AM, anti-ANT antibody, CypD CRISPR activation plasmid, and Protein A/G beads were from Santa Cruz Biotechnology (Shanghai, China); JC-1, DAPI, and Calcium Green 5N were from Invitrogen Molecular Probes (Beijing, China); Annexin V- FITC/ PI apoptosis detection kit was purchased from Vazyme Biotech (Shanghai, China). HRP, Alexa Fluor 488, Alexa Fluor 594-conjugated secondary antibody, and mitochondria isolation kit were purchased from Thermo Scientific (Shanghai, China).

1.2 Preparation and quality control of Mitochondria

The mouse livers were homogenized in mitochondria isolation buffer (250 mM Sucrose, 10 mM Tris-Mops, 0.1 mM EGTA-Tris, 0.1% BSA; pH 7.4) and stored on ice. The homogenates were centrifuged in 1.5 ml tubes at 2800 rpm for 10 min at 4 ℃. The supernatant was then centrifuged at 8800 rpm for 10 min, and the resulting pellet was washed with 1 ml of mitochondria buffer (without BSA) followed by another centrifugation at 8800 rpm for 10 min. Isolation of rat brain mitochondria was achieved using a discontinuous Percoll gradient according to the method of Sims and Anderson (Sims and Anderson, 2008), with slight modification. The cortical tissue was dissected out on ice and stored in ice-cold isolation buffer (320 mM sucrose, 1 mM EGTA, 10 mM Tris-Base; pH 7.4). Approximately 500 mg of the tissue was manually homogenized in 12 % Percoll in ice-cold isolation buffer (10 % wt/vol) using a 2 ml Kontes Teflon homogenizer. The homogenate was layered on a gradient of 26 %

and 40 % Percoll in ice-cold isolation buffer. The mixture was then centrifuged at 30,700 rpm for 10 min to yield a dense fraction in the interface between the 26 % and 40 % Percoll layers. It was then diluted to 1:4 in ice-cold isolation buffer, washed, and centrifugated at 16,700 rpm for 12 min. A final washing step was performed with ice-cold isolation buffer and BSA (1 mg/ml) in an Eppendorf microcentrifuge at 7300 rpm for 5 min. The entire isolation procedure was performed on ice, and the mitochondria were used in experiments within 2 h of the decapitation of animals. To estimate the quality of the mitochondria, the respiratory control ratio (RCR) was tested by the methods previously reported by Marcu et al (Marcu et al., 2012).

1.3 Docking Methods

In silico Study. The binding of the GA to Human Cyclophilin D (CypD) was analyzed using AutoDockTools 1.5.6 and AutoDock Vina 1.1.2 docking programs.

Preparation of CypD Protein. The starting 3D structure of CypD was obtained from Protein Data Bank (PDB ID: 2BIT) and the crystallographic water molecules were eliminated. Then, the polar hydrogen atoms were added using the AutoDockTools 1.5.6.

Preparation of GA. The 3D structure of GA was generated by Pymol 0.99, and rotatable bonds were defined using AutoDockTools 1.5.6.

Molecular Docking. To explore the binding sites and interaction of GA on CypD, blind docking was carried out with the grid size set to 108, 102 and 120 Å in X, Y, Z dimensions respectively, with 1.00 Å grid spacing. The center of the grid was set to 12.082, 13.651 and 21.913. Thirty ligand-receptor complex conformations were generated, and the conformation with lowest binding free energy was considered for further analysis. The interaction of GA in the binding sites was then analyzed by AutoDockTools 1.5.6.

1.4 In vivo Experimental design and model assessment

The *in vivo* experiment in this study was composed of three parts. The first part was designed to test the effect of GA on the sensitivity of MPTP in isolated liver mitochondria. The second part aimed to assess the effects of GA on MCAO-induced cerebral mitochondrial damage. The third part was designed to determine the mechanisms of GA suppressing the sensitivity of MPTP following MCAO insult.

Part I. The effect of GA on the sensitivity of MPTP in isolated liver mitochondria

***Experiment I*: ** Ten male C57BL/6 mice, weighing 18-22 g, were randomly assigned to two groups.

Group I: The isolated liver mitochondria were divided into five samples in each mouse, pre-treated with control buffer, GA (0.1, 1, 10 μM) and CsA (1 μM), respectively, and then used to CRC assays. CRC of the control group was defined as CRC_0, other groups' were expressed as CRC_i. The ratio of CRC_i and CRC_0 were used for statistical analysis. Group II: Repeat the Group I test.

***Experiment II*: ** Ten male C57BL/6 mice, weighing 18-22 g, were randomly assigned to two groups.

Group I: The isolated liver mitochondria were divided into four samples in each mouse, pre-treated with control buffer, and then used to modeling mitochondrial swelling by incubation with menadione at concentrations of 50, 100, 200 μM, respectively. Group II: Repeat the Group I test.

***Experiment III*: ** Ten male C57BL/6 mice, weighing 18-22 g, were randomly assigned to two groups.

Group I: The isolated liver mitochondria were divided into four samples in each mouse, pre-treated with control buffer, control buffer, GA (10 μM) and CsA (1 μM), respectively, and then used to mitochondrial swelling assays. Group II: Repeat the Group I test.

Experiment IV**:** Six male C57BL/6 mice, weighing 18-22 g, were randomly assigned to two groups.

Group I: The isolated liver mitochondria were divided into five samples in each mouse, pre-treated with control buffer, control buffer, GA (1, 10 μM) and CsA (1 μM), respectively. After calcium uptake, mitochondria were lysed for immunoprecipitation. Group II: Repeat the Group I test.

Experiment V**:** Twenty male C57BL/6 mice, weighing 18-22 g, were randomly assigned to two groups: control group and GA (100 mg/kg) group (n=10). The design for Experiment V was shown in **Fig. 1H**. Control mice were injected with vehicle. Mitochondrial swelling assays were performed on isolated mitochondria from adult C57BL/6 mouse liver after GA was administered intravenously by tail vein injection (100 mg/kg) once a day for 6 days. Finally, six samples in each group were lysed for Western Blot (n=6).

Part II. The effects of GA on MCAO-induced cerebral mitochondrial damage in rats

Experiment I**:** A total of thirty-two male SD rats, weighing 250-300 g, were randomly assigned to six groups: Control group (n=4), sham group (n=4), MCAO group (n=6), MCAO + Reperfusion 24 h group (n=6), MCAO + Reperfusion 48 h group (n=6), MCAO + Reperfusion 72 h group (n=6). The mean arterial blood pressure, glucose, and heart rate were measured at 30 min before the insertion of the suture, insertion of the suture, and in the initial 30 min of reperfusion, and maintained within normal ranges in each of the experimental animal **(Supplementary Tab. 2)**

The following exclusion criteria were applied during the experiment (Dirnagl et al., 2010):

-Mortality of animals;

-No stroke, the rat with neurological deficits score of 0 point will be excluded; (The method of neurological deficits evaluation is according to Longa et al (Longa et al., 1989). Score 0: No apparent

neurological deficits; Score 1: Contralateral forelimb flexion; Score 2: Decreased resistance to lateral push; Score 3: Spontaneous movement in all directions and contralateral circling when pulled by tail; Score 4: did not walk spontaneously and had depressed levels of consciousness)

-Problems during induction of MCAO (excessive bleeding, prolonged operation time<30 min, thread placement). After model assessment, 24 male SD rats were used in the experiment (n=4). All animals were humanely sacrificed under anesthesia, and used for Western Blot.

***Experiment II*:** A total of one hundred and seven male SD rats, weighing 250-300 g, were randomly assigned to seven groups: Control group (n=4), sham group (n=20), sham + GA (50 mg/kg) group (n=4), sham + CsA (10 mg/kg) group (n=4), MCAO group (n=25), MCAO + GA (50 mg/kg) group (n=25), MCAO + CsA (10 mg/kg) group (n=25). The design for Experiment II is shown in **Supplementary Fig. 3**. The mean arterial blood pressure, glucose, regional cerebral blood flow (rCBF), and heart rate were measured at 30 min before the insertion of the suture, insertion of the suture, and in the initial 30 min of reperfusion, and maintained within normal ranges in each of the experimental animal **(Supplementary Tab. 3)**. The rCBF distal to the occlusion dropped to 20 % in the each groups and returned to pre-occlusion levels to the same extent after filament withdrawal **(Supplementary Fig. 4A)**. After model assessment, 92 male SD rats were used as following. Control group (n=4), sham group (n=20), sham + GA (50 mg/kg) group (n=4), sham + CsA (10 mg/kg) group (n=4), MCAO group (n=20), MCAO + GA (50 mg/kg) group (n=20), MCAO + CsA (10 mg/kg) group (n=20). All animals were humanely sacrificed 48 h following reperfusion under anesthesia. Four rats in each group were used for Western Blot. Eight rats in sham, MCAO, MCAO + GA (50 mg/kg), and MCAO + CsA (10 mg/kg) group were used for TTC staining. Similarly, four rats in above four group were used for TEM assay, and the remaining four rats were used to H & E staining and Immunohistochemistry.

Part III. The mechanisms of GA suppress the sensitivity of MPTP following MCAO insult

A total of eighty male SD rats, weighing 250-300 g, were randomly assigned to seven groups: sham group (n=12), sham + GA (50 mg/kg) group (n=4), sham + U0126 (30 mg/kg) group (n=4), MCAO group (n=15), MCAO + GA (50 mg/kg) group (n=15), MCAO + U0126 (30 mg/kg) group (n=15), and MCAO + GA (50 mg/kg) + U0126 (30 mg/kg) group (n=15). The design for Part III is shown in **Fig. 6G**, and the methods of rCBF and physiological parameters were identical to Part II- Experiment II. The rCBF distal to the occlusion dropped to 20 % in the each groups and returned to pre-occlusion levels to the same extent after filament withdrawal **(Supplementary Fig. 4B)**. Physiological parameters were maintained within normal ranges in each experimental animal **(Supplementary Tab. 4)**. After model assessment, 68 male SD rats were used as following. Sham group (n=12), sham + GA (50 mg/kg) group (n=4), sham + U0126 (30 mg/kg) group (n=4), MCAO group (n=12), MCAO + GA (50 mg/kg) group (n=12), MCAO + U0126 (30 mg/kg) group (n=12), and MCAO + GA (50 mg/kg) + U0126 (30 mg/kg) group (n=12). All animals were humanely sacrificed 48 h following reperfusion under anesthesia. Four rats in each group were used for Western Blot. Eight rats in sham, MCAO, MCAO + GA (50 mg/kg), MCAO + U0126 (30 mg/kg), and MCAO + GA (50 mg/kg) + U0126 (30 mg/kg) group were used for TTC staining.

References

Dirnagl, U. (2010). Standard operating procedures (SOP) in experimental stroke research: SOP for middle cerebral artery occlusion in the mouse. *Nat. Preced. Npre.* **2010.3492.2.** doi:10.1038/npre.2012.3492.3

Longa, E.Z., Weinstein, P.R., Carlson, S., and Cummins, R. (1989). Reversible middle cerebral artery occlusion without craniectomy in rats. *Stroke*. 20, 84-91. doi: 10.1161/01.STR.20.1.84

Marcu, R., Neeley, C.K., Karamanlidis, G., and Hawkins, B.J. (2012). Multi-parameter measurement of the permeability transition pore opening in isolated mouse heart mitochondria. *J. Vis. Exp. Pii.* **4131**. doi: 10.3791/4131

Sims, N.R., and Anderson, M.F. (2008). Isolation of mitochondria from rat brain using Percoll density gradient centrifugation. *Nat. Protoc*. 3, 1228-1239. doi:10.1038/nprot.2008.105

2. Supplementary Figures and Tables

2.1 Supplementary Figures

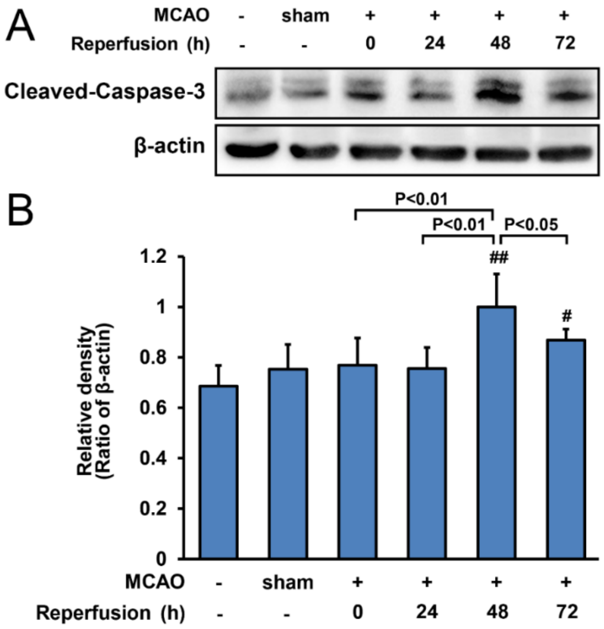

Supplementary Fig. 1 Cleaved-Caspase-3 overexpression following MCAO injury. Upon transient cerebral ischemia injury, the levels of Cleaved-Caspase-3 were upregulation robust and rapid. Immunoblot, β-actin loading control. Data are reported as the means ± S.D. (n=4). ## P<0.01 *versus* sham group.

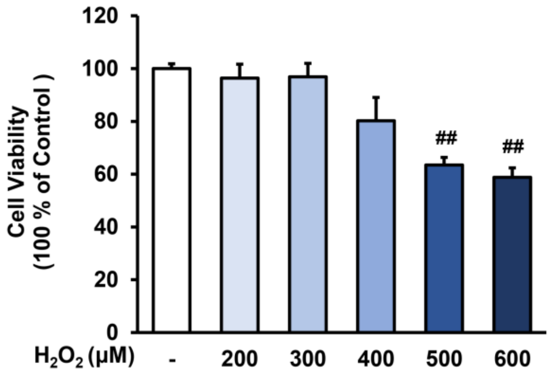

Supplementary Fig. 2 Concentration-dependent effect of H_2O_2 induced-cytotoxicity in SH-SY5Y cells. Cells were exposed to different concentrations of H_2O_2 for 4 h. The cell viability was assessed by an MTT assay. Data are reported as the means ± S.D. (n=5). Non-treated cells served as controls. ## P<0.01 *versus* control group.

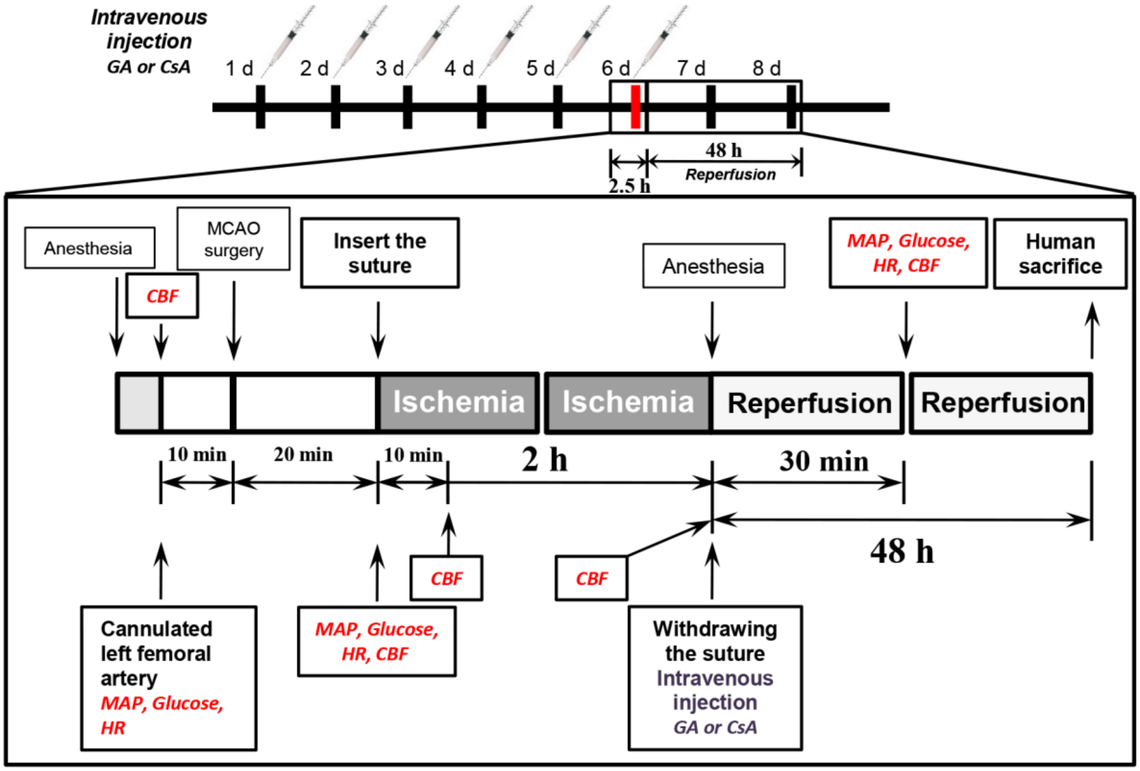

Supplementary Fig. 3 Schematic of the design for Part II-Experiment II of the study.

GA or CsA was administered intravenously once a day for 5 days respectively. The rats were anesthetized with chloral hydrate (350 mg/kg intraperitoneal injection). The left femoral artery was cannulated; blood pressure and heart rate were measured throughout the study by Power Lab System (AD Instruments, Australia). The level of rCBF was achieved using a laser-Doppler flowmeter (Moor Instruments, UK). Focal cerebral ischemia was induced by MCAO. The GA and CsA-treated group was administered GA (50 mg/kg, intravenous injection) or CsA (10 mg/kg, intravenous injection) immediately after withdrawing the suture. To alleviate pain, animals received 0.05 mg/kg subcutaneous buprenorphine immediately after the reperfusion anesthesia. The rCBF, mean arterial blood pressure, glucose and heart rate were measured at 30 min before the insertion of the suture, insertion of the suture, and in the initial 30 min of reperfusion. All animals were humanely sacrificed 48 h following reperfusion under anesthesia.

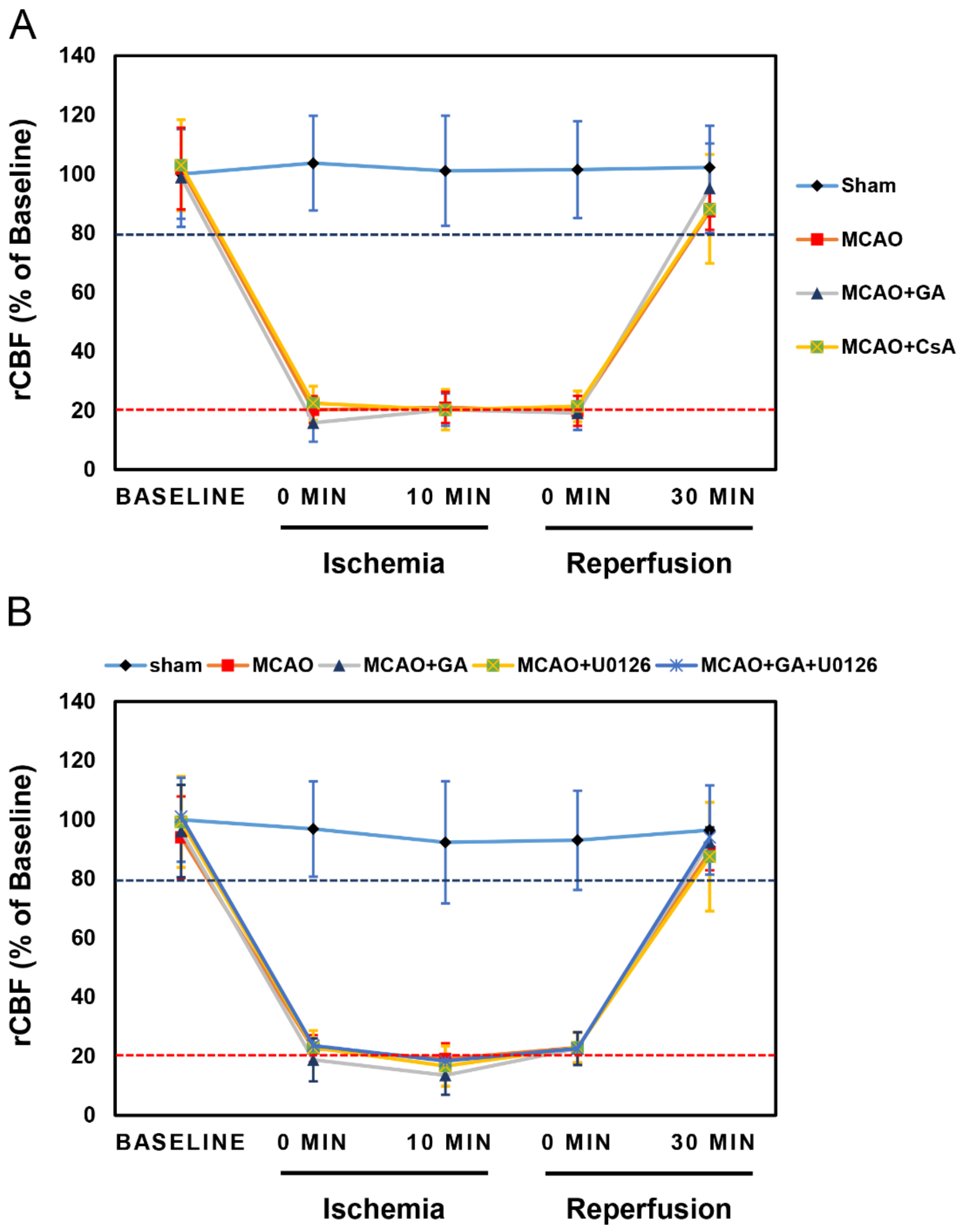

Supplementary Fig. 4 Changes of rCBF in MCAO rats.

The rCBF was reduced to 20 % of the pre-ischemic baseline value 10 min after ischemia and returned to the 80 % of baseline at 30 min after reperfusion. **(A)** The rCBF in Part II-Experiment II. Data are reported as the means ± S.D. (n=20). **(B)** The rCBF in Part III. Data are reported as the means ± S.D. (n=12).

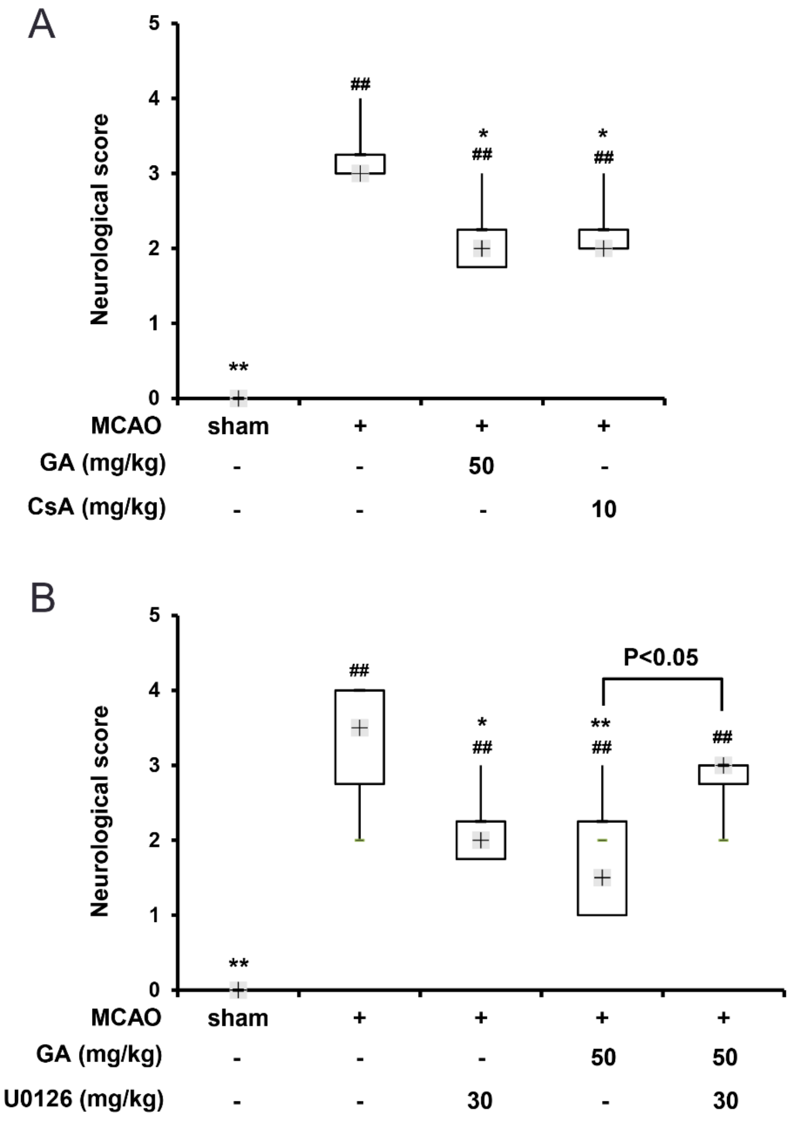

Supplementary Fig. 5 Observation of neurological deficits in each group.

No neurological deficits were observed in the sham treated rats, **(A)** The neurological score in Part II-Experiment II. Data are reported as the means ± S.D. (n=20). **(B)** The neurological score in Part III. Data are reported as the means ±S.D. (n=12). Neurological deficit scores were expressed as the median, and the statistical significance of the difference between groups was determined by a non-parametric Mann Whitney test. ## P<0.01 *versus* sham group. * P<0.05, ** P<0.01 *versus* MCAO group.

2.2 Supplementary Tables

Supplementary Tab. 1 CRC assay

Compand	Concentration (µM)	CRC_i/CRC_0 (%)[a]	SD[b]
**CsA**	1	334	22
	0.1	101	11
**GA**	1	148	19
	10	214	14

[a] Increase of the calcium retention capacity of mouse liver mitochondria after incubation with inhibitor.
[b] n =10.

Supplementary Tab. 2

Physiological parameters in Part II- Experiment I following MCAO surgery

Group		Weight (g)	MAP (mmHg)	HR (beats/min)	Glucose (mmol/L)
**Sham (n=4)**	**Baseline**	289 ± 11.2	99.1 ± 8.95	400 ± 18	6.2 ± 0.9
	**Ischemia**		98.2 ± 10.0	392 ± 21	5.8 ± 1.2
	**Recovery**		99.5 ± 8.5	389 ± 34	6.0 ± 0.9
**MCAO (n=6)**	**Baseline**	290 ± 14.1	98.5 ± 9.5	401 ± 25	5.9 ± 1.0
	**Ischemia**		97.2 ± 7.5	399 ± 10	4.8 ± 0.7
**MCAO 24 h Reperfusion (n=6)**	**Baseline**	288 ± 10.0	99.2 ± 10.0	385 ± 21	5.2 ± 1.2
	**Ischemia**		98.8 ± 10.5	388 ± 31	6.1 ± 1.0
	**Recovery**		99.2 ± 9.1	389 ± 29	5.5 ± 0.8
**MCAO 48 h Reperfusion (n=6)**	**Baseline**	275 ± 11.5	99.7 ± 11.2	379 ± 25	4.8 ± 1.1
	**Ischemia**		98.5 ± 8.5	385 ± 35	5.5 ± 1.2
	**Recovery**		99.8 ± 12.5	380 ± 20	5.9 ± 1.3
**MCAO 72 h Reperfusion (n=6)**	**Baseline**	291 ± 8.0	98.7 ± 10.5	372 ± 25	5.5 ± 1.1
	**Ischemia**		99.5 ± 9.5	385 ± 31	5.2 ± 1.6
	**Recovery**		98.5 ± 8.8	413 ± 13	5.8 ± 0.8

The results are expressed as the mean ± S.D. (n=4-6); MAP, mean arterial pressure; HR, heart rate. Baseline, 30 min before the insertion of the suture; Ischemia, insertion of the suture; Recovery, 30 min after the insertion of the suture.

Supplementary Tab. 3 Physiological parameters in Part II- Experiment II following MCAO surgery

Group		Weight (g)	MAP (mmHg)	HR (beats/min)	Glucose (mmol/L)
Sham (n=20)	Baseline	273 ± 10.1	98.8 ± 10.1	398 ± 21	4.9 ± 0.6
	Ischemia		97.4 ± 11.9	402 ± 18	5.1 ± 1.0
	Recovery		98.4 ± 10.5	387 ± 29	5.0 ± 0.6
Sham+GA (n=4)	Baseline	268 ± 11.5	98.9 ± 12.4	391 ± 28	4.8 ± 1.0
	Ischemia		96.9 ± 9.2	390 ± 18	5.0 ± 1.0
	Recovery		97.8 ± 11.2	411 ± 21	5.0 ± 0.5
Sham+CsA (n=4)	Baseline	281 ± 9.1	99.1 ± 8.1	397 ± 28	5.2 ± 1.0
	Ischemia		98.7 ± 10.0	412 ± 18	4.8 ± 1.0
	Recovery		98.8 ± 7.1	409 ± 25	4.9 ± 0.5
MCAO (n=25)	Baseline	272 ± 8.5	98.5 ± 9.5	412 ± 15	5.1 ± 1.2
	Ischemia		99.2 ± 8.5	409 ± 10	4.7 ± 1.1
	Recovery		99.2 ± 7.9	395 ± 29	4.8 ± 0.9
MCAO+GA (n=25)	Baseline	265 ± 9.5	98.5 ± 9.1	389 ± 22	5.0 ± 1.0
	Ischemia		97.8 ± 11.5	402 ± 19	4.7 ± 1.5
	Recovery		98.9 ± 7.8	390 ± 31	5.2 ± 1.0
MCAO+CsA (n=25)	Baseline	278 ± 9.5	99.8 ± 9.2	391 ± 15	5.1 ± 1.1
	Ischemia		98.7 ± 10.0	390 ± 28	4.7 ± 1.0
	Recovery		98.2 ± 10.6	388 ± 24	4.9 ± 0.8

The results are expressed as the mean ± S.D. (n=4-25); MAP, mean arterial pressure; HR, heart rate. Baseline, 30 min before the insertion of the suture; Ischemia, insertion of the suture; Recovery, 30 min after the insertion of the suture.

Supplementary Tab. 4 Physiological parameters in Part III following MCAO surgery

Group		Weight (g)	MAP (mmHg)	HR (beats/min)	Glucose (mmol/L)
Sham (n=12)	Baseline	288 ± 12.5	99.5 ± 9.5	384 ± 14	5.4 ± 1.4
	Ischemia		99.5 ± 8.2	382 ± 19	4.9 ± 1.8
	Recovery		98.9 ± 10.0	400 ± 20	5.1 ± 0.9
Sham+GA (n=4)	Baseline	276 ± 9.5	99.1 ± 9.0	400 ± 15	4.2 ± 1.0
	Ischemia		97.5 ± 9.5	398 ± 16	4.5 ± 0.9
	Recovery		99.1 ± 9.5	379 ± 15	5.5 ± 1.1
Sham+U0126 (n=4)	Baseline	281 ± 7.5	97.5 ± 7.5	391 ± 18	5.9 ± 1.0
	Ischemia		100.5 ± 11.5	401 ± 20	4.9 ± 0.5
	Recovery		99.5 ± 9.5	389 ± 15	5.3 ± 1.6
MCAO (n=15)	Baseline	290 ± 10.0	99.5 ± 9.5	400 ± 11	5.6 ± 1.0
	Ischemia		98.0 ± 8.0	401 ± 9	5.7 ± 1.2
	Recovery		99.5 ± 11.5	399 ± 21	4.8 ± 1.6
MCAO+GA (n=15)	Baseline	285 ± 11.0	97.5 ± 10.1	385 ± 24	5.2 ± 0.8
	Ischemia		99.9 ± 9.5	392 ± 21	5.7 ± 1.0
	Recovery		98.0 ± 8.5	385 ± 25	4.9 ± 0.8

**MCAO+U0126 (n=15)**	**Baseline**	295 ± 8.5	98.0 ± 9.0	389 ± 25	5.4 ± 0.7
	**Ischemia**		99.5 ± 7.8	403 ± 24	5.2 ± 1.5
	**Recovery**		99.4 ± 10.0	398 ± 31	4.8 ± 1.9
**MCAO+GA+U0126 (n=15)**	**Baseline**	288 ± 10.5	100.2 ± 10.5	399 ± 10	5.0 ± 1.0
	**Ischemia**		98.9 ± 9.5	410 ± 28	4.4 ± 1.8
	**Recovery**		99.8 ± 10.1	389 ± 19	5.6 ± 1.7

The results are expressed as the mean ± S.D. (n=4-15); MAP, mean arterial pressure; HR, heart rate. Baseline, 30 min before the insertion of the suture; Ischemia, insertion of the suture; Recovery, 30 min after the insertion of the suture.

3. The Ethics of Animal Experimentation

CERTIFICATION

Jiangsu University
Laboratory Animal Management Committee
03/06/2016

LABORATORY ANIMAL MANAGEMENT COMMITTEE JIANGSU UNIVERSITY

This file is used to certify that the laboratory animals and animal experiment procedures are ethical in manuscript with the title of "Desensitizing mitochondrial permeability transition by ERK-cyclophilin D axis contributes to the neuroprotective effect of gallic acid against cerebral ischemia/reperfusion injury". The serial number is UJS-20160012.

The Laboratory Animal Management Committee of Jiangsu University is responsible for overseeing the care and use of all vertebrate animals on campus, and composed of scientists, technicians, veterinarians, students, and members of the public. The committee reviews each proposal to use animals, and decides whether the project has valid scientific goals and whether it is ethical and humane. The committee insists the principle of the 3R's-Replacement of animals whenever possible with alternatives such as computer models or cells; Reduction in the number of animals used; and Refinement of techniques to make them as humane as possible.

All procedures in the manuscript were reviewed in advance by the Laboratory Animal Management Committee of Jiangsu University and also met the guidelines of the National Institutes of Health Guide for the Care and Use of Laboratory Animals.
